# Ant's Nest as a microenvironment: Distinct *Mucoromycota* (Fungi) community of the red wood ants' (*Formica polyctena*) mounds

**DOI:** 10.1002/ece3.70333

**Published:** 2024-10-09

**Authors:** Igor Siedlecki, Michał Kochanowski, Julia Pawłowska, Gabriela Reszotnik, Alicja Okrasińska, Marta Wrzosek

**Affiliations:** ^1^ Botanic Garden, Faculty of Biology University of Warsaw Warsaw Poland; ^2^ Institute of Evolutionary Biology, Faculty of Biology, Biological and Chemical Research Centre University of Warsaw Warsaw Poland; ^3^ Faculty of Agriculture and Ecology Warsaw University of Life Sciences Warsaw Poland

**Keywords:** ant–fungal interactions, microenvironments, *Mucoromycota*, nest microbiome, red wood ants, temperate forests

## Abstract

Many social insect species build nests, which differ from the surrounding environment and are often occupied by specific organismal communities. These organisms may interact mutualistically or parasitically with the nest‐builders, or simply co‐occur, being able to survive in these microenvironments. In temperate forests, red wood ants (e.g. *Formica polyctena*) are known to create distinct, highly developed nests, which consist of large, above‐ground mounds, built primarily out of plant matter collected from the forest litter. The microorganismal communities of such mounds remain understudied. As representatives of *Mucoromycota* fungi commonly engage in the decomposition process of the forest litter, they would be expected to occur in the mounds. However, it is still not known whether the *Mucoromycota* community of these ants' nests differ from the one of the surrounding forest litter. In order to distinguish mound‐associated taxa, we characterized *Mucoromycota* communities of *Formica polyctena* mounds and the surrounding forest litter. We sampled four sites, twice in a season. Sampled material was plated on agar media and emerging *Mucoromycota* colonies were identified based on their morphology. Fungal identification was further confirmed using DNA barcoding. In order to compare described communities, PERMANOVA test and non‐metric multidimensional scaling ordinations were used. To distinguish taxa associated with the mounds, multilevel pattern analysis was performed. Our results show that the *Mucoromycota* community of *Formica polyctena'*s mound differs from the community of the surrounding forest litter. While representatives of *Entomortierella lignicola* and *Absidia cylindrospora* clade were found to be associated with the mound environment, representatives of *Umbelopsis curvata* and *Podila verticillata‐humilis* clade were associated with forest litter, and were rarely present in the mounds. Our findings strongly suggest that the red wood ants' nest is a specific microenvironment in the temperate forest floor, which is a preferred microhabitat for the mound‐associated *Mucoromycota*, possibly adapted to live in proximity to ants.

## Graphical abstract

Many social insect species build nests, which strongly differ from the surrounding environment and are often occupied by specific organismal communities. We compared the Mucoromycota (Fungi) community of red wood ant's (*Formica polyctena*) mounds with the community of the surrounding forest litter. We show that the fungal community of mounds is specific and that representatives of *Entomortierella lignicola* and *Absidia cylindrospora* clade are associated with the mound environment.

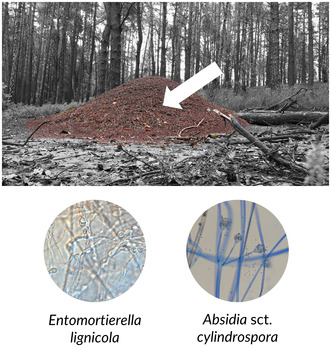

## 1 INTRODUCTION

The majority of ant species lead a social and stationary lifestyle, which includes active nest building (Hölldobler & Wilson, 1990). While forming their nests, ants highly modify their surroundings (De Almeida et al., 2020; Folgarait, 1998; Jouquet et al., 2006; Kovář et al., 2013; Meyer et al., 2013) and thus are often perceived as ecosystem engineers (Del Toro et al., 2012; Wills & Landis, 2018). In the environment, such actively maintained nests act as distinctive, environmental islands (Boots et al., 2012; Dauber et al., 2001; Folgarait, 1998), with their own specific and distinct bacterial (Boots et al., 2012; Lindström et al., 2019; Lucas et al., 2019; Song et al., 2023; Travanty et al., 2022) and animal communities (Hölldobler & Kwapich, 2022; Kronauer & Pierce, 2011; Parmentier et al., 2014; Wells et al., 2017). Some specifically adapted species, called obligate myrmecophiles, are even present exclusively in ants' nests (Hölldobler & Kwapich, 2022; Kronauer & Pierce, 2011; Parker & Grimaldi, 2014; Parmentier et al., 2014).

In the case of fungi, the number of studies that show a presence of distinct, fungal communities in ant‐made environments is still scarce (Boots et al., 2012; Brinker et al., 2019; Lindström et al., 2019; Lucas et al., 2019), with just a few examples of extensively studied mutualistic fungi present in ants' nests. Apart from fungus‐growing ants, with an extreme example of coevolution between leaf‐cutting ants and *Leucoagaricus gongylophorus* (Currie, 2001; Dejean et al., 2023; Hölldobler & Wilson, 1990; Mueller et al., 1998), fungal symbionts of ant nests have also been described for some ant species occupying domatia and for cardboard nest‐forming ants (Defossez et al., 2009; Nepel et al., 2014, 2016; Ruiz‐González et al., 2010). In the case of ‘carton ants’, ‘black‐yeasts’ were found to overgrow the walls of their nests and thus increase the strength of the whole structure (Dejean et al., 2023; Ruiz‐González et al., 2010). In all those ant‐fungal mutualistic interactions, while the beneficial roles of fungal partners differ, from nutritional to structural (Blatrix et al., 2012; Chomicki & Renner, 2017; Dejean et al., 2023; Hölldobler & Wilson, 1990; Ruiz‐González et al., 2010), the fungal presence always relies on the abundance and type of accumulated organic matter.

Interestingly, the nest‐mycobiota of the mound‐building *Formica*, which creates one of the most complex and long‐lasting, organic nests (Stockan & Robinson, 2016), has been severely understudied. The unique feature of their nest is a presence of an above‐ground mound built mostly out of dead plant material collected from the surroundings (Czechowski et al., 2012; Scherba, 1958; Stockan & Robinson, 2016, Figure 1). Until now, out of all mound‐building *Formica* species, only the mycobiota of *Formica exsecta* have been comprehensively described in a series of studies by Lindström et al. (2019, 2021, 2023). Those studies show that the mycobiome of *F. exsecta* ants' mounds is specific, more stable, more abundant, and significantly different from the mycobiome of surrounding forest soil. Additionally, representatives of *Ascomycota* (*Exophiala*, *Oidiodendron*, *Scleroconidioma*) and *Mucoromycota* (*Umbelopsis*) have been proposed as indicator taxa for the mound environment (Lindström et al., 2019). Similarly, in a study of Duff et al. (2016), the nest mycobiota of *F. ulkei* (a species closely related to *F. exsecta*) was shown to differ and be more abundant than the one in the surrounding soil. *Aspergillus navahoensis* and *A. pseudodeflectus* were found to be associated with the nest environment.

**FIGURE 1:**
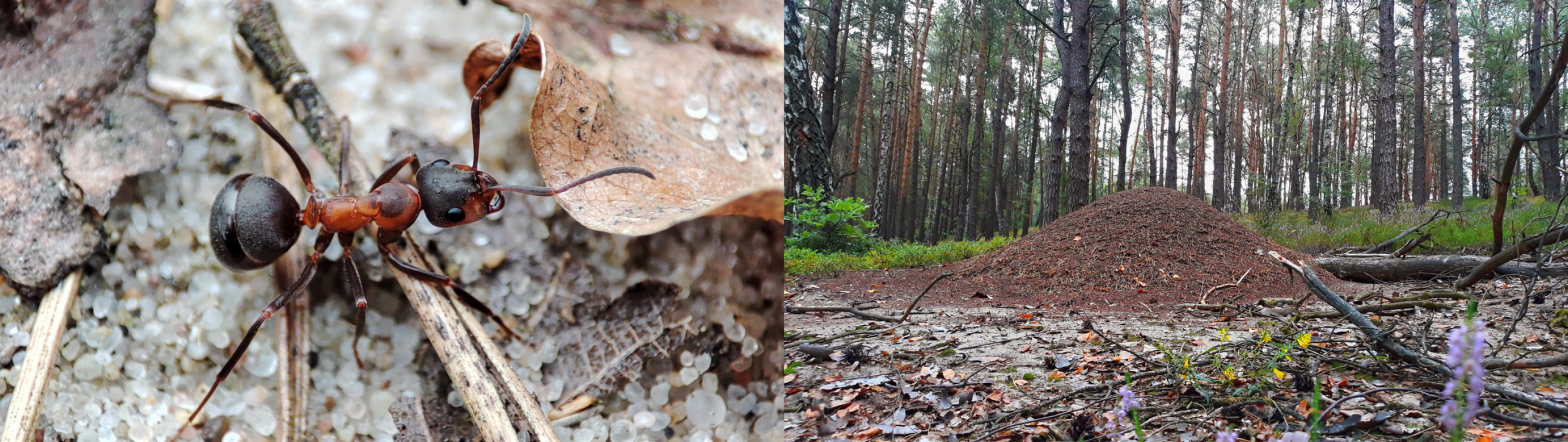
A worker of *Formica polyctena* ant and a mound of this species.

However, the knowledge about the mycobiota of the red wood ants (RWA; *Formica rufa* group as defined by Borowiec et al., 2021), another well‐known, monophyletic group of mound‐building *Formica*, remains limited. Representatives of this group are common in mixed and coniferous temperate forests, strongly affecting these ecosystems (Czechowski et al., 2012; Frouz & Jilková, 2008; Jílková, 2015; Jurgensen et al., 2008; Kilpeläinen et al., 2007; Stockan & Robinson, 2016). In the forest floor, they build large, domed, and long‐lasting mounds, which reach up to two meters in height and up to one cubic meter in volume (Czechowski et al., 2012; Stockan & Robinson, 2016, Figure 1). The most common organic components of those mounds are: pine needles, little twigs, pieces of bark, and other small plant parts. Additionally, mounds are usually located around a decaying stump (Castella et al., 2008; Czechowski et al., 2012; Frouz et al., 2016; Stockan & Robinson, 2016). Thanks to RWA's activity, physical and biochemical properties of their mounds significantly differ from the surrounding forest litter (Domisch et al., 2009; Frouz & Jilková, 2008; Frouz et al., 2016; Jílková, 2015; Kadochová, 2017; Kilpeläinen et al., 2007). Ants actively maintain stable microclimate within their nests regardless of the oscillating weather conditions. The temperature within the nest is usually kept between 15 and 32 centigrade during the active season and kept above the freezing point during winter (Frouz & Finer, 2007; Frouz et al., 2016). The nest is also usually slightly alkalized (Jílková et al., 2012), and has an increased amount of nutrients such as polysaccharides and simple sugars (Domisch et al., 2009; Frouz & Finer, 2007; Frouz et al., 2016; Kilpeläinen et al., 2007). When compared to the surrounding litter, an increased amount of antimicrobial substances, such as formic acid and coniferous resin is present in the mounds (Brütsch & Chapuisat, 2014; Brütsch et al., 2017; Castella et al., 2008; Christe et al., 2003). A large RWA mound can contain as much as 20 kg of tree resin (Christe et al., 2003). Due to ants' activity, there are also usually no plants growing on the mounds (Frouz & Jilková, 2008; Laakso & Setälä, 1998).

Importantly, the mound itself is not a homogenous environment. In the innermost parts of the mound, in comparison with the outer layer, lower daily and seasonal amplitude of temperatures are noted (Frouz, 2000), with the temperature in the center of the mound rarely dropping below the freezing point through the entire winter (Frouz et al., 2016). Additionally, higher terpene and resin concentrations are noted within the interior of the mound (Brütsch & Chapuisat, 2014; Sorvari & Hartikainen, 2021). Moisture within the mound also differs, with the surface layer being usually more moist than the interior part (Elo et al., 2018). Finally, the surface layer contains plant matter of smaller sizes than the mound interior (Maavara et al., 1994).

The microbial biomass in the mounds is usually higher than in the surrounding soil (Golubev & Bab'eva, 1972; Laakso & Setälä, 1998; Maksimova et al., 2016). So far, only yeast communities of RWA's mounds have been analyzed. Golubev and Bab'eva (1972) and Maksimova et al. (2016) have shown that yeast communities from ants' nests differ from yeast communities of the surrounding soil. In both of these studies, representatives of *Debaryomycetaceae* (*Debaryomyces hansenii*, *Schwanniomyces polymorphus*, and *S. vanrijiae*) were shown to be associated with ants' nests. Moreover, differences in yeast community composition were also observed within the nest, with a higher abundance of yeasts, but a lower species diversity of the yeast community found deeper in the mound in comparison to the surface layer (Maksimova et al., 2016).

Increased simple sugars concentrations noted in the RWA's mounds, would suggest that also representatives of *Mucoromycota* (as defined in Naranjo‐Ortiz & Gabaldón, 2019) often referred to as ‘sugar fungi’, should be expected in this specific environment. Similar to RWA, diverse *Mucoromycota* representatives are commonly present in soil, litter, rhizosphere, as well as in dead wood of coniferous and mixed temperate forests (Bahnmann et al., 2018; Carreiro & Koske, 1992; Gorfer et al., 2021; Grantina et al., 2012; Kwaśna et al., 2016; Osono et al., 2006; Summerbell, 2005; Tedersoo et al., 2014; Toju & Sato, 2018). They are also known to be engaged in the decomposition of coniferous needles (Millar, 2012; Osono et al., 2006), which are one of the main building materials of RWA's mounds.

However, detailed distribution, diversity, and ecology of *Mucoromycota* are still understudied (Naranjo‐Ortiz & Gabaldón, 2019; Voigt et al., 2021), with many species from this phylum remain undiscovered (Naranjo‐Ortiz & Gabaldón, 2019; Tedersoo et al., 2014). In recent years, many new species of fungi or new examples of fungal symbiosis have been described from insect‐made environments such as termite nests (Nel et al., 2021), ants carton nests (Voglmayr et al., 2011), plant domatia (Baker et al., 2017), and bark and ambrosia beetles galleries (Skelton et al., 2018). In the case of *Mucoromycota*, species diversity of many of such environments still remains unexplored (Naranjo‐Ortiz & Gabaldón, 2019; Voigt et al., 2021). Examples of *Mucoromycota* representatives associated with insects are known (Voigt et al., 2021; Zhu et al., 2022), including fungi from the *Entomortierella* genus, whose name etymology refers to insect‐association (Vandepol et al., 2020). However, the actual role of those fungi is still not well understood. Scarce available data suggest *Mucor hiemalis* and *Mucor fragilis* can be entomopathogenic (Bibbs et al., 2013; Zhu et al., 2022), whereas *Actinomortierella* aff. *ambigua* seems to be non‐pathologically associated with the fungivorous millipede (*Brachycybe lecontii*) (Macias et al., 2019). As the majority of described *Mucoromycota* species are easily cultivable and the DNA barcodes reference database for *Mucoromycota‐*type species is broad (Gherbawy et al., 2010; Vu et al., 2019; Wagner et al., 2013; Walther et al., 2013), *Mucoromycota* diversity in specific habitats could easily be studied with the use of culture based methods supplemented with molecular identification.

In recent years, some *Mucoromycota* fungi were isolated from RWA‐associated substrates (Clark, 2002; Hyde et al., 2017; Siedlecki et al., 2021). Representatives of the insect‐associated *Entomortierella* genus have been isolated from the content of infrabuccal pockets of *F. rufa* (Clark, 2002). In a study focused on the mycobiota of *F. polyctena* ants, strains of *Mucor*, *Entomortierella*, and *Absidia* were noted (Siedlecki et al., 2021). Moreover, *Mortierella formicae* has also been isolated and described from a cadaver of *F. polyctena* ant (Hyde et al., 2017).

Knowing about the common presence of *Mucoromycota* in the temperate forest litter, isolating *Mucoromycota* strains from RWA workers, and being aware of specific properties of RWA's mounds, we hypothesize the existence of a distinct *Mucoromycota* community occurring in this ant‐made environment. Further, we hypothesize the existence of *Mucoromycota* species, preferentially or exclusively present in the mounds. To test it, we describe and compare the *Mucoromycota* community present in mounds of *F. polyctena* (RWA) with the *Mucoromycota* community of the surrounding forest litter. To characterize fungal communities, we used a culture‐based approach. The isolates were preliminary classified based on their morphology and their identification was confirmed by rDNA barcoding. To distinguish taxa associated with the mounds, we performed a multilevel pattern analysis. Finally, we discuss why this specific, ant‐made environment could work as a preferred microhabitat for some *Mucoromycota*, possibly adapted to cohabit with ants.

## 2 MATERIALS AND METHODS

### 2.1 Study object

The ant *Formica polyctena* is one of the most common species belonging to RWA, a sister species to *F. rufa*, occurring in coniferous and mixed temperate forests (Borowiec et al., 2021; Czechowski et al., 2012). Within mound‐building *Formica* ants, *F. polyctena* makes the largest polydomous colonies made up of big, long‐lasting nests, housing even over one million individuals and reaching three meters in diameter (Czechowski et al., 2012; Stockan & Robinson, 2016). Whole supercolony of this species can consist of up to 400 million individuals (Stockan & Robinson, 2016). Nests of *F. polyctena* are usually located in more shaded spots compared to other RWA species (Czechowski et al., 2012; Stockan & Robinson, 2016).

### 2.2 Study material collection

**FIGURE 2:**
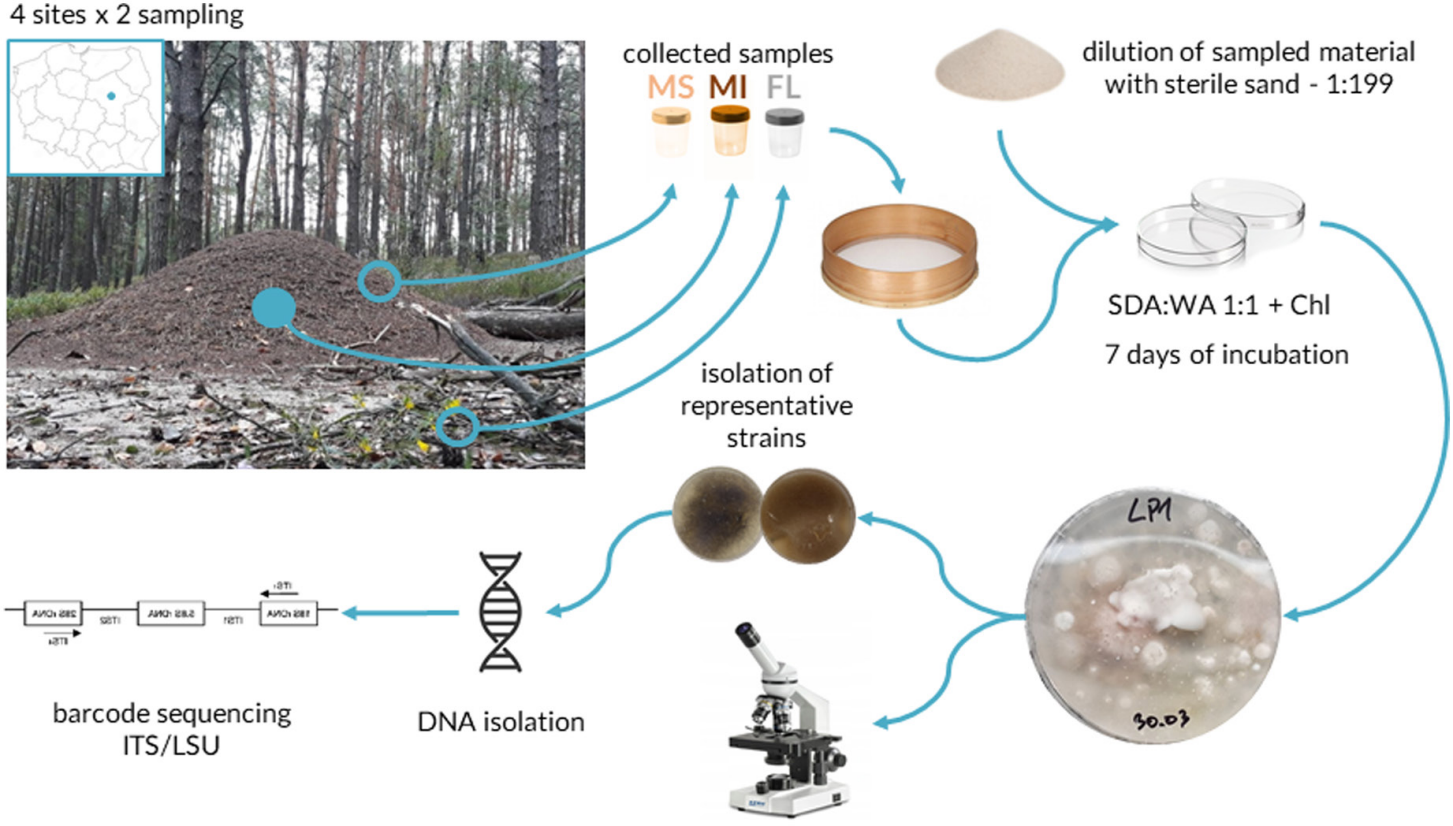
Study scheme. The blue point on the map indicates the sampling area. The photo shows one of the *Formica polyctena* mounds sampled for the study. The blue dot on the photo of the mound indicates sampling from the depth of 15 cm, and the blue rings indicate sampling of the substrate surface (5 cm depth). Chl, chloramphenicol; FL, forest litter; MI, mound interior; MS, mound surface; SDA, Sabouraud Dextrose Agar; WA, Water Agar.

The collection of study material was conducted twice, on the 22nd of August and the 5th of October in 2020, in the pine forests of Mazovian Voivodeship (Poland). Material was collected from four different sites separated by at least 15 km. Each time, sampling at each site included a collection of three different substrates: two nest substrates (mound surface—MS and mound interior—MI) and forest litter—FL (Figure 2). Four 25 mL subsamples from four different parts of the mound surface were pooled in order to obtain one 100 mL MS sample. For MI samples, 100 mL of mound material was excavated at a depth of 15 cm from the central part of the mound. For FL samples, 25 mL of litter from a forest floor was collected at four distinct spots surrounding the mound and merged to obtain one 100 mL FL sample. Each collection spot for the FL was located at least 10 meters away from the mound. The material was collected into sterile, plastic containers and transported on ice to the laboratory, where it was stored at −20°C until further analysis. In total, 24 samples were collected (8 FL, 8 MS, and 8 MI). Ants were identified as *Formica polyctena* before the sampling. Ant colony identification was based on the morphology of five workers per nest, using Czechowski et al. (2012) key. Ant's nest material was collected under the permission of The Regional Directorate for Environmental Protection (RDOŚ) in Warsaw. Permit number: WPN‐I.6401.428.2020.PK.2. More metadata on sampling and sampled mounds is available in Table A of Appendix.

### 2.3 Isolation of *Mucoromycota*

For each collected sample, the material was first sifted through a 1 mm sieve. The sifted material was then mixed with sterile sand until final dilution of 1:199, following Warcup's soil plate method, with a modification by Mańka (1964) for topsoil, and then plated in sterile conditions with 10 replicates. Each replicate contained 0,1 g of diluted material and was evenly distributed onto Petri dishes (90 mm) containing twice‐diluted Sabouraud Dextrose Agar medium (SDA:WA, 1/1), with added chloramphenicol (25 μg/mL). The inoculated plates were placed in dark conditions and incubated at 18°C for 7 days. After 7 days, all fungal colony forming units (CFUs) were counted for each plate.

### 2.4 Identification of isolates

Only *Mucoromycota* representatives were selected for further studies. The isolates were assigned to taxonomic units based on molecular and morphological characteristics. Macroscopic features of the fungal colonies were observed using a stereoscopic microscope (“Nikon SMZ 800” and EOS camera) and strains' micromorphology was studied on slides stained using lactophenol cotton blue under a light microscope (“Nikon ELIPSE Ni” and Nikon DS—Ri 2 camera with NIS Elements software). For sporulating *Mucoromycotina* (as defined in Naranjo‐Ortiz & Gabaldón, 2019), all grown colonies sharing similar morphology were grouped into distinct morphotypes (Chalabuda, 1973; Skirgiełło et al., 1979; Watanabe, 2002). To avoid the omission of cryptic species, for each morphotype, up to three representative strains (one per studied substrate) were preserved as axenic cultures. For *Mortierellomycotina* (as defined in Naranjo‐Ortiz & Gabaldón, 2019) and non‐sporulating *Mucoromycotina*, axenic cultures were preserved for all grown colonies.

Total genomic DNA was extracted from all preserved axenic cultures using the ExtractMe Genomic DNA Kit (Blirt S.A., Gdańsk, Poland), according to the manufacturer's protocol. Molecular identification of the isolates was based on commonly used fungal barcodes: the internal transcribed spacer region (ITS) or the large subunit nuclear ribosomal DNA (LSU) (Liu et al., 2012; Schoch et al., 2012; Vu et al., 2019). Primarily the ITS marker was amplified using a protocol described in Okrasińska et al. (2021), and primer pairs ITS1f and ITS4 (White et al., 1990). However, due to high variability of this region in some *Mucoromycota* fungi (Walther et al., 2013), we were unable to obtain ITS sequence for some strains. In these cases, the large subunit nuclear ribosomal DNA (LSU) marker was amplified, using a protocol described in Siedlecki et al. (2023), and primers pairs: LROR (Rehner & Samuels, 1994) and LR5 (Vilgalys & Hester, 1990) or NL1 and NL4 (O'Donnell, 1993). PCR products were purified with the ExtractMe DNA Clean‐Up & Gel‐Out kit (Blirt S.A.) and sequenced using the Sanger method by an external company, Genomed S.A. (Warsaw, Poland). Forward and reverse sequences were assembled using the DNA Subway software (Williams et al., 2014).

Obtained consensus sequences were compared with data available in NCBI GenBank (ncbi.nlm.nih.gov accessed on 28 December 2023) using the BlastN algorithm (Altschul et al., 1990). The species‐level names were assigned when morphology based identification was in line with top blast hits (refined to type strains and reference strains deposited in recognized public culture collections), meeting the following three criteria: (1) sequence coverage >90%, (2) sequence identity >97% for ITS and >98% for LSU, and (3) differentiation from the closest species >0,7% for ITS and >0,3% for LSU. Otherwise, the term “species clade” or higher level taxonomic rank was used to name isolated taxonomic units.

### 2.5 Statistical analysis

Analysis and data visualization were performed using R v4.1.2 in RStudio (R Core Team, 2020; RStudio Team, 2020). Differences between the study treatments in: a *Mucoromycota* abundance (counted as number of CFUs), and taxa richness were tested using ANOVA with a post‐hoc Tukey test. The Shannon diversity index was calculated using a vegan package (Oksanen et al., 2019). Differences in Shannon diversity indexes between the study treatments were tested using ANOVA with a post‐hoc Tukey test. In order to assess the differences in *Mucoromycota* community composition between the substrates, sampling sites, and collection months, permutational multivariate analysis of variance was performed with Bonferroni adjustment using adonis function, Bray–Curtis distance and 999 permutations (Oksanen et al., 2019). Homogeneity of variance within and between groups was tested using betadisper function from vegan package (Oksanen et al., 2019). Non‐metric multidimensional scaling ordinations (Bray–Curtis distances) were used to visualize community compositions. A Wisconsin double standardization was performed during NMDS analysis to reduce the signal of abundantly grown species. Species significantly shaping the composition of *Mucoromycota* communities (*p* < .05) displayed on the ordination plot were analyzed by the envfit (permutations = 999) function within the package vegan (Oksanen et al., 2019). In order to indicate taxa associated with a specific substrate, Multilevel pattern analysis (MPA) was conducted using the indicspecies package (Cáceres & Legendre, 2009), both correlation indices and indicator value functions were used in the analysis. Figures were generated with ggplot2 (Villanueva & Chen, 2019).

## 3 RESULTS

In the study, we isolated 4800 colony forming units (CFUs) from 24 samples: eight mound interior (MI), eight mound surface (MS), and eight forest litter (FL) (Table B of Appendix). 27.1% of all isolated colonies were assigned as *Mucoromycota* (*MM*) representatives (1301 *MM* CFUs), 1.38% colonies remained unidentified, and the rest (71.52%) were assigned as non‐*Mucoromycota*. After assignment to morphotypes, 162 colonies were preserved as axenic strains (Table C of Appendix). Forty‐seven strains represented 17 morphotypes of sporulating *Mucoromycotina* and the rest were strains of *Mortierellomycotina* and non‐sporulating *Mucoromycotina*. The ITS sequence was obtained for 106 strains, and the LSU sequence for 56 strains. Based on the morphological and molecular data, all *MM* colonies obtained in this study were assigned to 23 separate taxonomic units (Table C of Appendix), which encompassed four families (*Cunninghamellaceae*, *Mortierellaceae*, *Mucoraceae*, *Umbelopsidaceae*) and nine genera (*Absidia*, *Entomortierella*, *Gongronella*, *Gryganskiella*, *Linnemannia*, *Mortierella*, *Mucor*, *Podila*, *Umbelopsis*).

**FIGURE 3:**
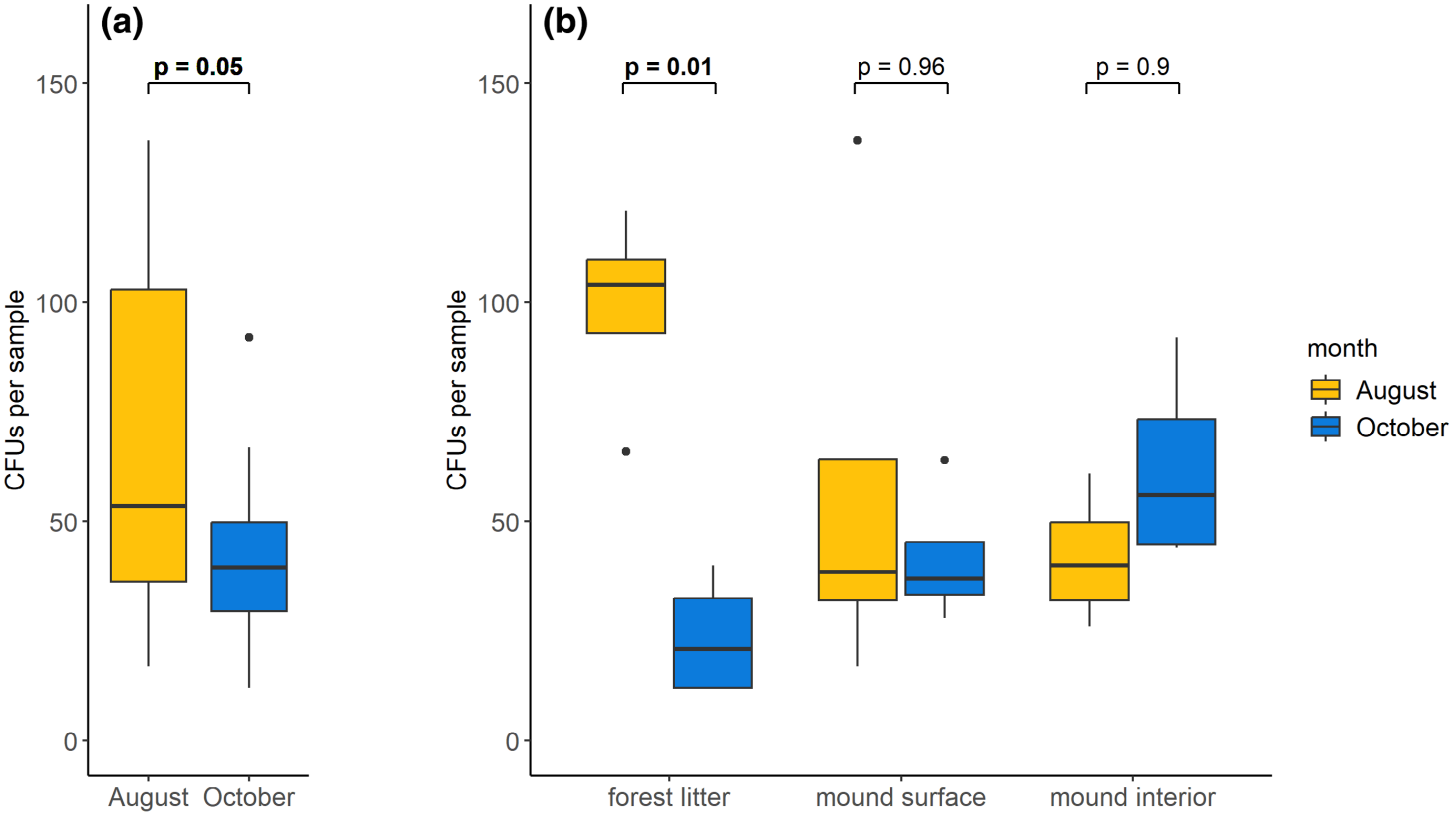
The averaged abundance of *Mucoromycota* regarding different sampling months (a), and sampled substrates and months combined (b). The boxplots cover values between the first and third quartile, the middle line represents the median number of *MM* CFUs per study variant, the whiskers represent maximum and minimum values below the upper and lower fence, and points represent outliers. The *p*‐value shown above the boxplots is an outcome of ANOVA with post‐hoc Tukey HSD tests, *p* <= .05 is bolded.

The number of isolated *MM* CFUs did not differ significantly between studied substrates (ANOVA, *p* = .68, *F* = 0.39) and accounted on average for 48.67 CFUs (sd = 29.36) per sample. On average, more *MM* CFUs were isolated from the August than the October samples (ANOVA, *p* = .05, *F* = 4.39) (Figure 3a). However, on the substrate level, such seasonal differences were observed only for the FL substrate (ANOVA, *p* = .01, *F* = 6.03; Tukey HSD Test, *p* = .01) (Figure 3b). We did not observe such a difference for the mound samples (Figure 3b).

**FIGURE 4:**
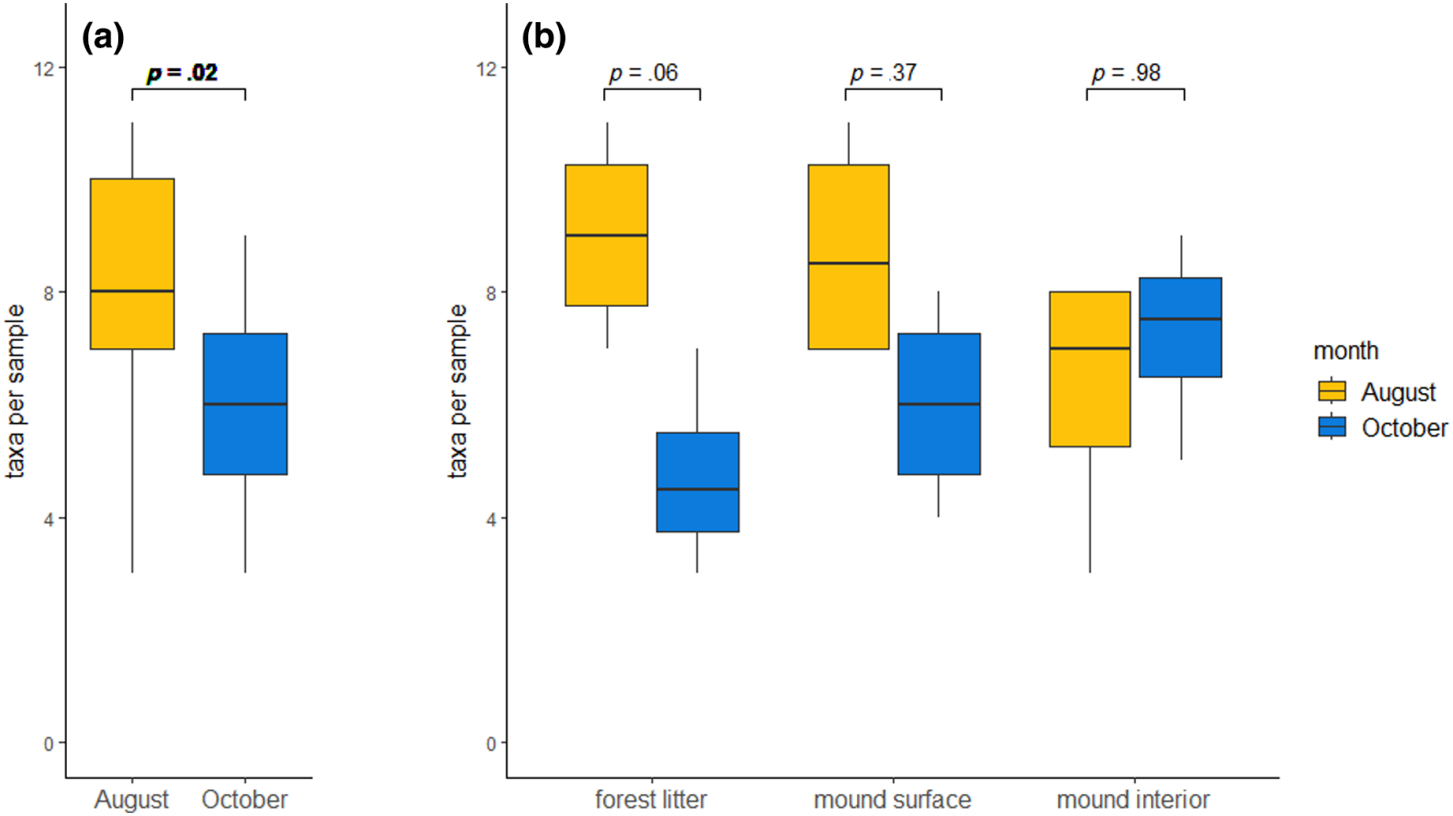
The averaged *Mucoromycota* taxa diversity regarding different sampling months (a), and sampled substrates and months combined (b). The boxplots cover values between the first and third quartile, the middle line represents the median number of *MM* taxonomic units per study variant, and the whiskers represent maximum and minimum values below the upper and lower fence. The *p*‐value shown above the boxplots is an outcome of ANOVA with post‐hoc Tukey HSD tests, *p* < .05 is bolded.

On average, 6.71 *MM* taxa (SD = 2.13) were noted for each studied sample. Between studied substrates, no significant differences were observed in taxa richness (ANOVA, *F* = 0.24, *p* = .793), nor in the Shannon diversity index (ANOVA, *F* = 0.23, *p* = .80). As for the CFUs abundance, more taxa were isolated in August than in October (ANOVA, *F* = 6.49, *p* = .021) (Figure 4a). Similarly, a higher Shannon index was noted for the August samples (av = 1.39, SD = 0.34) than the October ones (av = 1.14, SD = 0.35), however, this result was not significant (ANOVA, *F* = 3.27, *p* = .08). On the substrate level, although the seasonal difference was the biggest in case of the forest litter substrate (Figure 4b), it was not statistically significant (ANOVA, *p* = .04, *F* = 3.93; Tukey HSD Test, *p* = .06).

**FIGURE 5:**
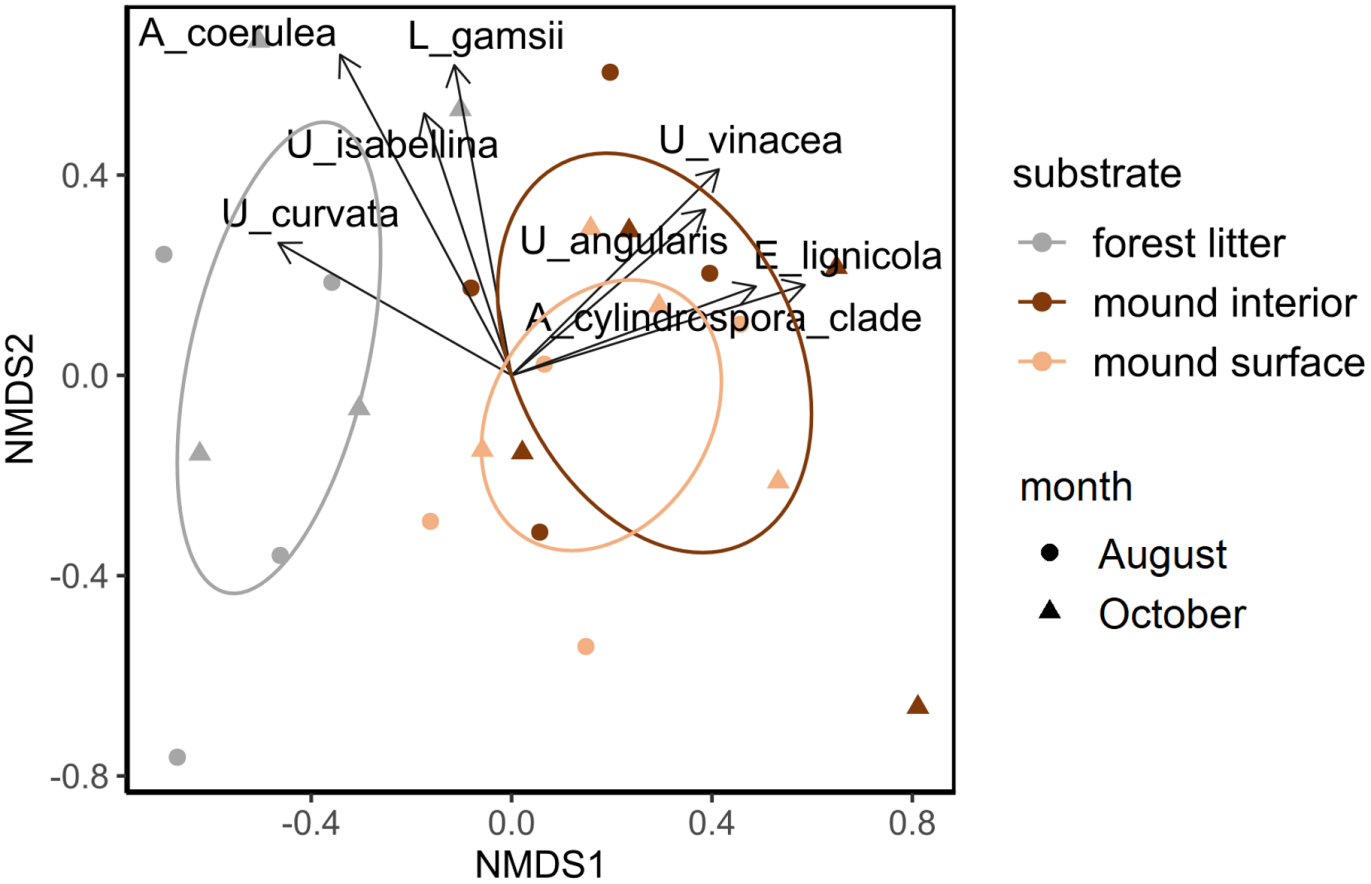
Ordination of *Mucoromycota* communities obtained from studied samples, which is the result of two‐dimensional ordination using NMDS, based on the Bray–Curtis dissimilarity matrix, computed from 24 taxa matrices transformed by Wisconsin double standardization. The colors of the points represent different substrates, and their shapes represent different months. Vectors represent species significantly shaping the composition of *MM* communities (*p* < .05). Ellipses indicate 95% confidence intervals around centroids of different substrates.

The *MM* community composition differed significantly between the substrates (PERMANOVA, *F* = 3.14, *p* = .002, Figure 5). Post‐hoc tests showed significant differences between mound interior communities and forest litter communities (pairwise adonis, *p*.adj = .042). NMDS ordination graph for *MM* communities recorded for different substrate samples shows three clusters: forest litter, mound surface, and mound interior clusters (Figure 5). While the mound surface and mound interior clusters are highly overlapping, the forest litter cluster is distinct. Analysis suggests *Absidia cylindrospora* clade (sensu Zong et al., 2021), *Entomortierella lignicola, Umbelopsis vinacea*, and *U. angularis* as taxa associated with the mound and *U. curvata* as taxon associated with the forest litter (Figure 5).

In both, correlation indices and indicator values analyses, *Entomortierella lignicola* was shown to be associated with the mound material, both with the surface and the interior of the mound (MPA, *r* = 0.69, *p* < .01; IV = 0.96, *p* < .01). Additionally, in the correlation indices analysis, *Absidia cylindrospora* clade was shown to be associated with the interior of the mound (MPA, *r* = 0.62, *p* < .01) and *U. curvata* with the forest litter (MPA, *r* = 0.47, *p* = .043). In the indicator values analysis, *Podila verticillata‐humilis* clade (sensu Vandepol et al., 2020) turned out to be associated with forest litter (MPA, IV = 0.72, *p* = .02).

**FIGURE 6:**
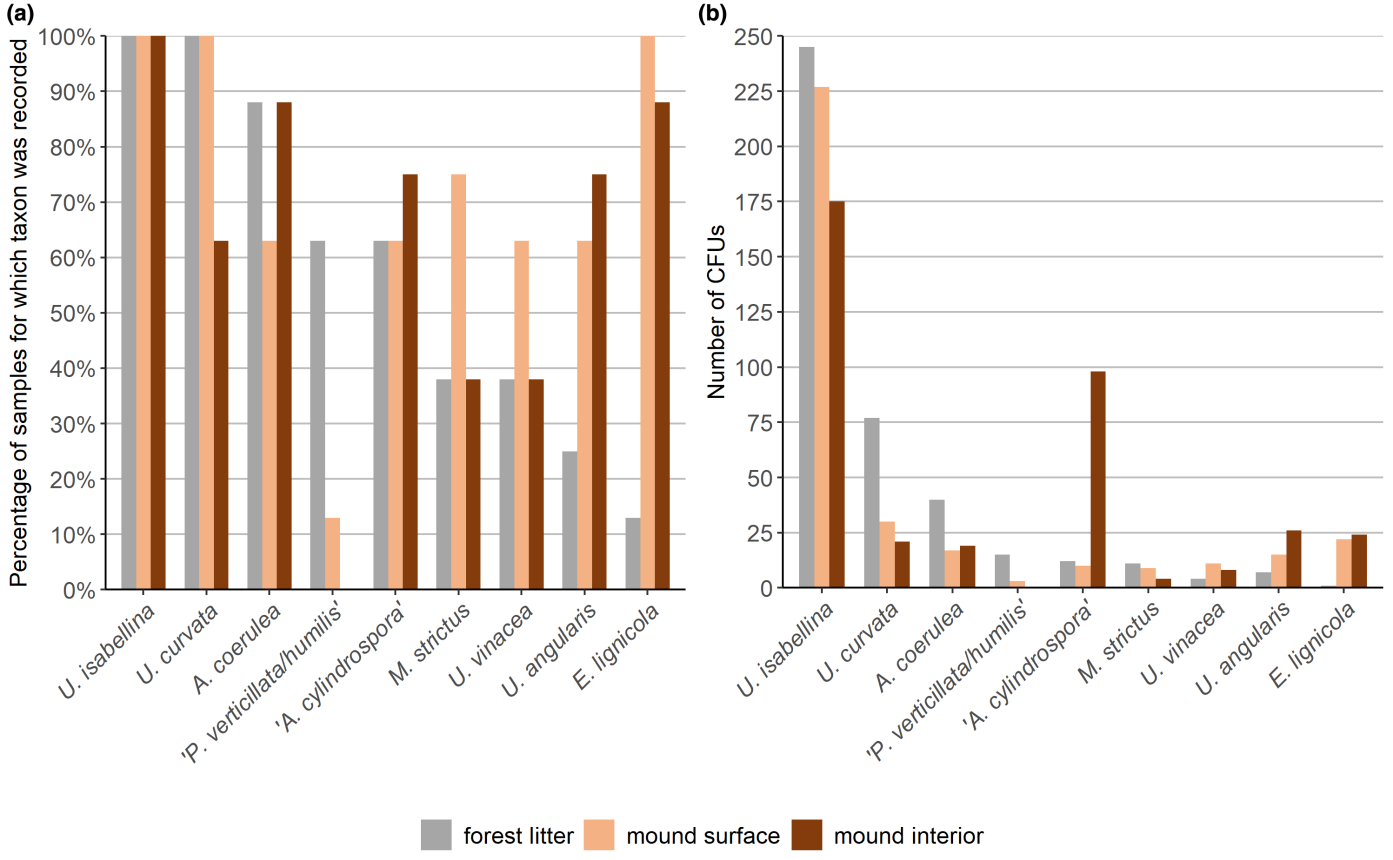
Taxa occurrence in the study. (a) Prevalence of taxa according to isolation substrate. Only taxa that were isolated from at least 50% of samples of any substrate are displayed on the graph. (b) Number of CFUs of taxa isolated in the study regarding isolation substrate. Taxa which were isolated less than 10 times are not displayed. In both graphs: Taxa are arranged in descending order for the forest litter substrate, and “*A. cylindrospora*” and “*P. verticillata/humilis*” mean respectively representatives of *A. cylindrospora* clade and *P. verticillata‐humilis* clade.

The most commonly isolated were representatives of the genus *Umbelopsis*. *U. isabellina* was the most abundant species (50% of *MM* CFUs), which was recorded from all of the studied samples (Figure 6a). Representatives of *U. curvata* were recorded more often from FL and MS samples, and representatives of *U. angularis* were more prevalent in the mound environments (Figure 6a). Commonly isolated were also representatives of *Absidia*, with *A. coerulea* being as prevalent in FL as in MI samples, and *A. cylindrospora* being more abundant inside the nest (82% of this taxa CFUs) (Figure 6b). Within *Mortierellacae*, representatives of *E. lignicola* were prevalent in the mound substrates (100% MS and 88% MI samples), where they were occurring almost exclusively (98% of this taxa CFUs) (Figure 6b). On the other hand, strains of *Podila verticillata‐humilis* clade were isolated more frequently from the forest litter (88% of this taxa cfu) (Figure 6b).

## 4 DISCUSSION

*Mucoromycota* is a phylum grouping some of the basal fungal lineages (Naranjo‐Ortiz & Gabaldón, 2019). Although the knowledge on this group is constantly growing (e.g. Gryganskyi et al., 2023; Muszewska et al., 2021; Pawłowska et al., 2019), until now, very few papers focus on their ecological requirements and mode of life (e.g. Telagathoti et al., 2021), especially in the case of invertebrate–fungal interactions (e.g. Nguyen et al., 2023).

Our study shows that representatives of *Mucoromycota* were present in the insect‐made environment, in particular that *Mucoromycota* communities formed on the surface and in the interior of *Formica polyctena*'s mounds were similarly abundant and diverse as *Mucoromycota* communities of the surrounding forest litter. However, while the fungal communities isolated from the August forest litter samples were more diverse and more abundant than communities isolated from the corresponding October samples, such a result was not observed in case of the mound samples (Figures 3 and 4). Even though most of the *Mucoromycota* taxa were isolated from each of the studied substrates (Figure 6), we observed a clear distinction between the *Mucoromycota* communities of the mound and of the surrounding forest litter (Figure 5). While strains of *Umbelopsis isabellina* were common in all studied substrates, representatives of *Entomortierella lignicola* were mound‐associated, and representatives of *Podila verticillata/humilis* clade were litter‐associated (Figure 6a). Additionally, representatives of *Umbelopsis curvata* were more often isolated from the forest litter samples than from the mound samples (Figure 6b). Although the taxonomic composition of *Mucoromycota* communities found in the mound interior and the mound surface was similar, representatives of the *A. cylindrospora* clade were abundantly isolated only from the mound interior (Figure 6b).

As representatives of *Mucoromycota* are common in forest litter (Gorfer et al., 2021; Qu et al., 2021; Tedersoo et al., 2014), they are most likely transferred into the mound together with plant material collected by ants. Representatives of *Mucoromycota* were also part of the fungal community described for the mostly organic mounds of *Formica exsecta* (Lindström et al., 2019, 2021). On the contrary, in the study of Duff et al. (2016) in which the mycobiota of *F. ulkei* nests was analyzed, strains of *Mucoromycota* (then classified as Zygomycota) were isolated in very low numbers and were excluded from further analysis. Additionally, antimicrobial substances such as resin and formic acid commonly present in RWA's mounds are known to decrease fungal growth (Brütsch & Chapuisat, 2014; Brütsch et al., 2017; Christe et al., 2003). Similar numbers of *Mucoromycota* CFUs isolated from mound and litter samples suggest that fungi from this group may have developed some way of handling these antimicrobial substances. Further studies using growing assays of the mound‐isolated *Mucoromycota* in the presence of resin and formic acid could help verify this hypothesis.

There were no significant differences between *Mucoromycota* mound communities (both in the number of CFUs and the diversity of *Mucoromycota*) between August and October sampling. This could be a result of more stable environmental conditions (especially temperature) occurring in the ant‐made environment throughout those months than in surrounding litter (Frouz & Finer, 2007; Frouz et al., 2016). Our results are in line with the results of Lindström et al. (2021) study in which more stable microbial communities were observed in mounds of *F. exsecta* throughout seasons and years, strengthening the hypothesis that nests of mound‐building *Formica* could act in the forest floor as a reservoir for microbial taxa less tolerant of climatic fluctuations (Lindström et al., 2021). Further studies that would include winter sampling while describing the RWA mounds' mycobiota could help verify this hypothesis.

Although *Mucoromycota* are commonly thought to be saprotrophic “sugar fungi” with scarce enzymatic repertoire (Botha & Botes, 2014), only recently several researches are unrevealing their broader physiological capacities, opening floor for further discussion on the role they may play in the ants' nests environments. *Mucoromycota* fungi are used as saccharification agents and in fermentation processes (Chen, 2021), their proteases are used in the food industry (Yegin et al., 2011), and lipases of some mucoralean representatives can act in fatty acids, oils and fats modification, processes such as hydrolysis of glycerides, transesterification, and esterification (Rodrigues & Fernandez‐Lafuente, 2010). At the same time, they quickly colonize suitable substrata, as their growth is very fast in comparison with ascomycetous and basidiomycetous fungi (Kirk, 1999). In consequence, they potentially can change the properties of the “food” particles as soon as it is introduced into the nest. Moreover, *Mucoromycota* fungi are well known for their capacity to produce fatty acids (e.g. linoleic acid) (Mohamed et al., 2019; Sokołowska et al., 2023). Interestingly, these are shown to be precursors of some sex hormones in lepidopteran representatives (Herrera et al., 2019). Further studies on physiology of fungi co‐occurring with ants can improve our understanding of insect‐fungi interactions in this specific environment.

Specific physical and chemical properties of RWA mounds such as adjusted temperature, increased pH, and accumulation of nutrients and resin, distinguish this ant‐made microenvironment from the surrounding forest litter (Christe et al., 2003; Domisch et al., 2009; Frouz & Jilková, 2008; Jílková et al., 2012). These properties most likely shape the specific mounds' Mucoromycota communities observed in our work. In previous studies, specificity of fungal communities of ants' mounds was also observed for the mounds of other mound‐building Formica: *F. aquilonia*, *F. exsecta*, and *F. ulkei* (Duff et al., 2016; Lindström et al., 2019; Maksimova et al., 2016). In the first two studies: of Duff et al. (2016), and of Maksimova et al. (2016), similarly as in our case, a culture‐based approach was used, and in both studies, the analyzed mycobiota was specific and more abundant than fungal communities in the corresponding non‐nest soil. In Lindström and others' metabarcoding study (Lindström et al., 2019) of the microbiome of *F. exsecta* mounds, the mound microbiome was more abundant, and significantly different from the mycobiome of surrounding forest soil. Together with those other studies, our results strengthen the mound's ecosystemic distinctiveness hypothesis. In the case of *F. polyctena*, population stability, large colony size, and connectivity of mounds in polydomous colonies made by this species (Czechowski et al., 2012; Stockan & Robinson, 2016), may result in even stronger stability and specificity of their mycobiota. Possibly, dispersive propagules of the mound‐associated fungal taxa could migrate between separate mounds in the polydomous colony on ants' cuticle or in their infrabuccal pockets, without the necessity to survive in the external environment. However, while a diverse fungal community was previously isolated either from cadavers of *F. polyctena* (Siedlecki et al., 2021) or from their infrabuccal pockets (Siedlecki et al., 2022), there were no studies yet which would show if representatives of Mucoromycota could be transported by ants.

Highly overlapping mound inside and mound surface clusters in NMDS analysis (Figure 5) suggest that despite some differences being noted between those substrates (Brütsch & Chapuisat, 2014; Elo et al., 2018; Frouz et al., 2016; Maavara et al., 1994; Sorvari & Hartikainen, 2021), they are not large enough to differentiate *Mucoromycota* communities present in those microenvironments. However, on the level of individual taxa, we observed a higher prevalence of litter‐associated taxa (*P. verticillata/humilis* clade and *U. curvata*) in the mound surface than in the mound interior, suggesting that the mound surface could work as an ecotone between the litter and the interior of the mound (Figure 6). Possibly, future studies focused on analyzing fungal communities in different layers of the mound during winter and spring, could reveal bigger differences between the communities of the different mound layers, as large differences in temperature between the surface and inside part of the mound are observed during those seasons (Frouz & Finer, 2007; Frouz et al., 2016).

Fungi from the *Umbelopsis* genus are known as common coniferous forest litter dwellers. They are often isolated from decaying coniferous needles, roots, bark, and wood (Käaeik & Kennebfelt, 1957; Kuhlman, 1969; Kwaśna et al., 2016; Meredith, 1960; Osono et al., 2006; Sewell, 1959; Söderström, 1975), as well as root endophytes of coniferous trees (Hoff et al., 2004; Kernaghan & Patriquin, 2011; Rim et al., 2021; Terhonen et al., 2014). The similar abundance of *Umbelopsis* representatives in the mound material as in the litter suggests that available nutrients and conditions are good enough for a stable *Umbelopsis* presence in the *Formica polyctena* nests. Omnipresence of *Umbelopsis* shown in our study somewhat contradicts the findings of Lindström et al. (2019). In their study, the genus *Umbelopsis* was listed as a core taxon for the *Formica exsecta* mound environment. Possible explanations encompass different methodological approaches, as only molecular methods were used in Lindström's study, or some unknown environmental differences between studied litter.

Even though we did not observe a strong preference towards any of the studied substrates for the whole *Umbelopsis* genus, we did observe such preferences on the species level. While strains of *U. isabellina* were commonly isolated from all substrate types, *U. curvata* occurred more often in the forest litter. As representatives of *U. isabellina* are commonly isolated from fresh or decaying wood of coniferous trees (Fisher et al., 1991; Giordano et al., 2009; Holdenrieder & Sieber, 1992; Holdenrieder et al., 1994; Kuhlman, 1969; Linnemann, 1941; Sewell, 1959), their abundant presence in the mound may also be related with the usual presence of a decaying tree stump in the base part of the aboveground part of the nest (Stockan & Robinson, 2016). However, as *U. curvata* have been distinguished from *U. ramanniana* quite recently (Wang et al., 2022), it is difficult to compare our findings with older studies on the ecology of *Umbelopsis* spp. and thus understand the observed preferences of this species. More extensive studies on the litter of temperate forests with an increased sampling of *F. polyctena* mounds, linked together with ecological experiments on *Umbelopsis* species, could explain better the specificity and adaptation mechanisms in the observed pattern.

Species of *Mortierellaceae* are known to be common in temperate coniferous forest soil and litter (Qu et al., 2021; Santalahti et al., 2016; Tedersoo et al., 2014), found also within fungal communities decomposing coniferous needles (Osono et al., 2003), and wood (Behnke‐Borowczyk et al., 2018; Fukasawa et al., 2011; Mäkipää et al., 2017). In our study, while representatives of *Mortierellaecae* were found in all studied environments, preferences towards certain microenvironments were observed on a lower taxonomic level. *Entomortierella lignicola* seems to be strongly associated with the mound environment, and representatives of *Podila verticillate‐humilis* clade occur more often in the forest litter. The most plausible explanation of this variability could be the difference in pH level between studied microenvironments. Previous study by Jílková et al. (2012) analyzing the pH of wood ant mounds showed that the pH in the mound is typically higher than that of the surrounding forest soil, and we speculate that a similar difference could occur in the case of our analyzed substrates. Furthermore, Linnemann (1941) stated that the basic factor determining the occurrence of *Mortierellaceae* species was the soil pH (later confirmed in studies of Enghusen (1956) and Turner and Pugh (1961)). As both *Podila humilis* and *P. verticillata* were characterized by Chalabuda (1973) as species commonly isolated from acidic forest soil and litter (preferred pH 4.0–4.1 for *P. verticillata*, and pH 3.7–6.3 for *P. humilis*), higher pH usually observed in the mounds could be the reason for the observed decreased abundance of *P. verticillata‐humilis* strains in the ant‐made environment. Additionally, as species of *P. verticillata‐humilis* clade are known to coexist with *Pinus* mycorrhiza (Chalabuda, 1973; Oh et al., 2019), their observed smaller abundance in the mounds could also be related to reduced amounts of alive plant roots in this microenvironment (Frouz & Jilková, 2008; Laakso & Setälä, 1998).

*Entomortierella lignicola* (previously *Mortierella lignicola*) is a species commonly isolated from decaying bark and wood, as well as soils, mostly forest ones (Giordano et al., 2009; Kuhlman, 1969; and based on metadata in GBIF and CBS culture collection). Therefore, the presence of fungi from this species in mounds is most probably related to the material of which the mounds are made. However, such a large difference in *E. lignicola* abundance between the litter and the mound suggests that ants actively stimulate the growth of this fungus or that the properties of the ant‐made microhabitat particularly favor the development of this species' representatives. A hypothesis of adaptation towards the insect‐made environments could be partially supported by previous isolation of *E. lignicola* from decayed wood with termite nests (Watanabe et al., 1998) and from the cadavers of *F. polyctena* ants (Siedlecki et al., 2021). Moreover, this species, belonging to the *M. lignicola* clade (sensu Wagner et al., 2013), is closely related to species also previously isolated from *Camponotus* and *Formica* ants and those ants' infrabuccal pockets (Clark, 2002; Hyde et al., 2017; Siedlecki et al., 2021). Importantly, *E. lignicola*, *E. beljakovae*, and *Mortierella formicae* were also found in the infrabuccal pockets of *F. polyctena* (Siedlecki et al., 2022). Interestingly, all those ant‐associated species are known to abundantly produce enlarged cells filled with oil droplets called gemmae (Wagner et al., 2013). We thus hypothesize that mycelium of this fungi could serve as a supplementary, nutrient‐rich food source for ants, as also suggested for gemmae‐producing *Actinomortierella* sp. aff *ambigua* and fungivorous millipede (Macias et al., 2019). However, further studies focused on ants' interactions with *Entomortierella* spp. are needed to verify those hypotheses.

*Absidia* fungi are found in forest soil, rhizosphere, and litter (Hesseltine & Ellis, 1964; Söderström, 1975), as well as in decomposing coniferous needles (Brandsberg, 1969), roots, and wood (Hesseltine & Ellis, 1964; Kuhlman, 1969). We thus expected representatives of this genus to be evenly distributed across all studied substrates. Therefore, the preference of *Absidia cylindrospora* clade representatives towards the interior of the mound is a surprising finding. Possibly higher temperatures in the mounds could allow for more abundant growth of these fungi as most species of *Absidia cylindrospora* clade are mesophilic, with the optimum growth temperature between 25°C and 34°C (Hoffmann, 2010; Hoffmann et al., 2007). Moreover, Klamer et al. (2001) isolated some strains from this clade from compost piles, where environmental conditions are similar to the ones in mounds. Additionally, few species belonging to this clade, are known to be insect‐related, with *A. psychrophilia* found in mycangia of ambrosia beetles (Hesseltine & Ellis, 1964) and *A. cylindrospora* together with *A. spinosa* isolated from the nests of *Aphaenogaster texana carolinensis* ants (Zettler et al., 2002). These results, when put together with our findings, suggest that representatives of this clade are adapted to survive in the proximity of ants.

Our results suggest that the mound community of *Mucoromycota* consists mostly of saprotrophic fungi commonly occurring in coniferous forest litter. However, the nest community is characterized by an increased abundance of *Entomortierella lignicola* and strains belonging to *Absidia cylindrospora* clade, and decreased abundance of *Umbelopsis curvata* and *Podila verticillata/humilis* clade. These findings suggest that *F. polyctena* ants' mound could serve as a preferred microhabitat for the mound‐associated species which could be otherwise repressed by other microorganisms present in the forest litter. However, which specific mound properties are the ones promoting the growth of these fungi mostly remain to be characterized, as well as the possible effect of the mound‐associated *Mucoromycota* on the ant colonies.

## AUTHOR CONTRIBUTIONS

**Igor Siedlecki:** Conceptualization (lead); data curation (lead); formal analysis (supporting); funding acquisition (equal); investigation (lead); methodology (lead); supervision (supporting); visualization (supporting); writing – original draft (lead); writing – review and editing (supporting). **Michał Kochanowski:** Data curation (supporting); formal analysis (lead); investigation (supporting); methodology (supporting); visualization (lead); writing – original draft (supporting). **Julia Pawłowska:** Methodology (supporting); writing – review and editing (lead). **Gabriela Reszotnik:** Investigation (supporting). **Alicja Okrasińska:** Writing – review and editing (supporting). **Marta Wrzosek:** Funding acquisition (equal); investigation (supporting); methodology (supporting); supervision (lead); writing – original draft (supporting); writing – review and editing (supporting).

## FUNDING INFORMATION

The study was financially supported by the statutory funds of the University of Warsaw Botanic Garden, and by the Ministry of Science and Higher Education through the University of Warsaw intramural grants “IV.4.1. A complex programme of support for UW PhD students – microgrants, 1st edition” in the “Excellence Initiative – Research University” program (BOB‐IDUB‐622‐32/2022). I.S. was supported by the Ministry of Science and Higher Education through the University of Warsaw intramural grants “IV.4.1. A complex program of support for UW PhD students – preparation of doctoral dissertations, 1st edition” in the “Excellence Initiative – Research University” program (BOB‐IDUB‐622‐115/2023).

## CONFLICT OF INTEREST STATEMENT

The authors declare no conflicts of interest.

## Supporting information


**Table A.** Study sites metadata.

**Table B.** Number of fungal CFUs isolated from samples.

**Table C.** List of representative strains.

**Table D.** Raw Data.

## Data Availability

Representative voucher specimens are stored in the Institute of Evolutionary Biology (University of Warsaw, Poland) collection (10% glycerol, −80°C) and the General Herbarium, University of Warsaw [WA] (dry specimens). Sequence data generated for this study are available in the GenBank database (http://www.ncbi.nlm.nih.gov/genbank). All reference numbers and taxon identification details are provided in Table B of Appendix 1. R script for statistical analysis is available online at https://github.com/mjkochanowski/mounds2024_v2. Raw data for the study are provided in Table D of Appendix 1.

## References

[ece370333-bib-0148] Altschul, S. F. , Gish, W. , Miller, W. , Myers, E. W. , & Lipman, D. J. (1990). Basic local alignment search tool. Journal of Molecular Biology, 215, 403–410. 10.1016/S0022-2836(05)80360-2 2231712

[ece370333-bib-0001] Bahnmann, B. , Mašínová, T. , Halvorsen, R. , Davey, M. L. , Sedlák, P. , Tomšovský, M. , & Baldrian, P. (2018). Effects of oak, beech and spruce on the distribution and community structure of fungi in litter and soils across a temperate forest. Soil Biology and Biochemistry, 119, 162–173. 10.1016/j.soilbio.2018.01.021

[ece370333-bib-0002] Baker, C. C. , Martins, D. J. , Pelaez, J. N. , Billen, J. P. , Pringle, A. , Frederickson, M. E. , & Pierce, N. E. (2017). Distinctive fungal communities in an obligate African ant‐plant mutualism. Proceedings of the Royal Society B: Biological Sciences, 284(1850), 20162501. 10.1098/rspb.2016.2501 PMC5360918 28298347

[ece370333-bib-0003] Behnke‐Borowczyk, J. , Kwasna, H. , Kokot, K. , Haluszczak, M. , & Lakomy, P. (2018). Abundance and diversity of fungi in oak wood. Dendrobiology, 80, 143–160. 10.12657/denbio.080.014

[ece370333-bib-0004] Bibbs, C. S. , Vitoreli, A. M. , Benny, G. , Harmon, C. L. , & Baldwin, R. W. (2013). Susceptibility of Latrodectus geometricus (Araneae: Theridiidae) to a Mucor strain discovered in north central Florida, USA. Florida Entomologist, 96(3), 1052–1061. 10.1653/024.096.0344

[ece370333-bib-0005] Blatrix, R. , Djiéto‐Lordon, C. , Mondolot, L. , La Fisca, P. , Voglmayr, H. , & McKey, D. (2012). Plant‐ants use symbiotic fungi as a food source: New insight into the nutritional ecology of ant–plant interactions. Proceedings of the Royal Society B: Biological Sciences, 279(1744), 3940–3947. 10.1098/rspb.2012.1403 PMC3427587 22859596

[ece370333-bib-0006] Boots, B. , Keith, A. M. , Niechoj, R. , Breen, J. , Schmidt, O. , & Clipson, N. (2012). Unique soil microbial assemblages associated with grassland ant species with different nesting and foraging strategies. Pedobiologia, 55(1), 33–40. 10.1016/j.pedobi.2011.10.004

[ece370333-bib-0007] Borowiec, M. L. , Cover, S. P. , & Rabeling, C. (2021). The evolution of social parasitism in Formica ants revealed by a global phylogeny. Proceedings of the National Academy of Sciences, 118(38), e2026029118. 10.1073/pnas.2026029118 PMC8463886 34535549

[ece370333-bib-0008] Botha, A. , & Botes, A. (2014). Encyclopedia of food microbiology (II ed., pp. 834–840). Elsevier.

[ece370333-bib-0009] Brandsberg, J. W. (1969). Fungi isolated from decomposing conifer litter. Mycologia, 61(2), 373–381. 10.1080/00275514.1969.12018738

[ece370333-bib-0010] Brinker, P. , Weig, A. , Rambold, G. , Feldhaar, H. , & Tragust, S. (2019). Microbial community composition of nest‐carton and adjoining soil of the ant *Lasius fuliginosus* and the role of host secretions in structuring microbial communities. From Antagonism to Mutualism: The Chemical Basis of Insect‐Fungus Interactions, 38, 44–53. 10.1016/j.funeco.2018.08.007

[ece370333-bib-0011] Brütsch, T. , & Chapuisat, M. (2014). Wood ants protect their brood with tree resin. Animal Behaviour, 93, 157–161. 10.1016/j.anbehav.2014.04.024

[ece370333-bib-0012] Brütsch, T. , Jaffuel, G. , Vallat, A. , Turlings, T. C. , & Chapuisat, M. (2017). Wood ants produce a potent antimicrobial agent by applying formic acid on tree‐collected resin. Ecology and Evolution, 7(7), 2249–2254. 10.1002/ece3.2834 28405288 PMC5383563

[ece370333-bib-0013] Cáceres, M. D. , & Legendre, P. (2009). Associations between species and groups of sites: Indices and statistical inference. Ecology, 90(12), 3566–3574. 10.1890/08-1823.1 20120823

[ece370333-bib-0014] Carreiro, M. M. , & Koske, R. E. (1992). Effect of temperature on decomposition and development of microfungal communities in leaf litter microcosms. Canadian Journal of Botany, 70(11), 2177–2183. 10.1139/b92-269

[ece370333-bib-0015] Castella, G. , Chapuisat, M. , & Christe, P. (2008). Prophylaxis with resin in wood ants. Animal Behaviour, 75(4), 1591–1596. 10.1016/j.anbehav.2007.10.014

[ece370333-bib-0016] Chalabuda, T. V. (1973). Griby Roda Mortierella Coemans. Nauka.

[ece370333-bib-0017] Chen, P. (2021). Lactic acid bacteria in fermented food. In Advances in probiotics (pp. 397–416). Academic Press. 10.1016/B978-0-12-822909-5.00024-1

[ece370333-bib-0018] Chomicki, G. , & Renner, S. S. (2017). The interactions of ants with their biotic environment. Proceedings of the Royal Society B: Biological Sciences, 284(1850), 20170013. 10.1098/rspb.2017.0013 PMC5360932 28298352

[ece370333-bib-0019] Christe, P. , Oppliger, A. , Bancalà, F. , Castella, G. , & Chapuisat, M. (2003). Evidence for collective medication in ants. Ecology Letters, 6(1), 19–22.

[ece370333-bib-0020] Clark, D. W. (2002). Fungi associated with the infrabuccal pockets of Camponotus pennsylvanicus and other Formicine ants [Master's thesis]. University of Toronto, Department of Botany.

[ece370333-bib-0021] Currie, C. R. (2001). A community of ants, fungi, and bacteria: A multilateral approach to studying symbiosis. Annual Reviews in Microbiology, 55(1), 357–380. 10.1146/annurev.micro.55.1.357 11544360

[ece370333-bib-0022] Czechowski, W. , Radchenko, A. , Czechowska, W. , & Vepsäläinen, K. (2012). The ants of Poland with reference to the myrmecofauna of Europe. Natura Optima Dux Foundation.

[ece370333-bib-0023] Dauber, J. , Schroeter, D. , & Wolters, V. (2001). Species specific effects of ants on microbial activity and N‐availability in the soil of an old‐field. European Journal of Soil Biology, 37(4), 259–261. 10.1016/S1164-5563(01)01094-9

[ece370333-bib-0024] De Almeida, T. , Blight, O. , Mesléard, F. , Bulot, A. , Provost, E. , & Dutoit, T. (2020). Harvester ants as ecological engineers for Mediterranean grassland restoration: Impacts on soil and vegetation. Biological Conservation, 245, 108547. 10.1016/j.biocon.2020.108547

[ece370333-bib-0025] Defossez, E. , Selosse, M.‐A. , Dubois, M.‐P. , Mondolot, L. , Faccio, A. , Djieto‐Lordon, C. , McKey, D. , & Blatrix, R. (2009). Ant‐plants and fungi: A new threeway symbiosis. New Phytologist, 182(4), 942–949. 10.1111/j.1469-8137.2009.02793.x 19383109

[ece370333-bib-0026] Dejean, A. , Azémar, F. , Naskrecki, P. , Tindo, M. , Rossi, V. , Faucher, C. , & Gryta, H. (2023). Mutualistic interactions between ants and fungi: A review. Ecology and Evolution, 13(8), e10386. 10.1002/ece3.10386 37529578 PMC10375366

[ece370333-bib-0027] Del Toro, I. , Ribbons, R. R. , & Pelini, S. L. (2012). The little things that run the world revisited: A review of ant‐mediated ecosystem services and disservices (hymenoptera: Formicidae). Myrmecological News, 17, 133–146.

[ece370333-bib-0028] Domisch, T. , Finer, L. , Neuvonen, S. , Niemelä, P. , Risch, A. C. , Kilpeläinen, J. , Ohashi, M. , & Jurgensen, M. F. (2009). Foraging activity and dietary spectrum of wood ants (*Formica rufa* group) and their role in nutrient fluxes in boreal forests. Ecological Entomology, 34(3), 369–377. 10.1111/j.1365-2311.2009.01086.x

[ece370333-bib-0029] Duff, L. B. , Urichuk, T. M. , Hodgins, L. N. , Young, J. R. , & Untereiner, W. A. (2016). Diversity of fungi from the mound nests of *Formica ulkei* and adjacent non‐nest soils. Canadian Journal of Microbiology, 62(7), 562–571. 10.1139/cjm-2015-0628 27192606

[ece370333-bib-0030] Elo, R. A. , Penttinen, R. , & Sorvari, J. (2018). Distribution of oribatid mites is moisture‐related within red wood ant *Formica polyctena* nest mounds. Applied Soil Ecology, 124, 203–210. 10.1016/j.apsoil.2017.11.013

[ece370333-bib-0031] Enghusen, H. (1956). Bodenkundlich‐mykologische Studie an Stuttgarter Steppenschwarzerden im Vergleich mit Waldböden: (Mit 2 Lagepl. U. 1 Taf. Im Text).

[ece370333-bib-0032] Fisher, P. J. , Petrini, O. , & Petrini, L. E. (1991). Endophytic ascomycetes and deuteromycetes in roots of Pinus sylvestris. Nova Hedwigia (Germany, FR), 52(1), 11–15.

[ece370333-bib-0033] Folgarait, P. J. (1998). Ant biodiversity and its relationship to ecosystem functioning: A review. Biodiversity and Conservation, 7(9), 1221–1244. 10.1023/A:1008891901953

[ece370333-bib-0034] Frouz, J. (2000). The effect of nest moisture on daily temperature regime in the nests of *Formica polyctena* wood ants. Insectes Sociaux, 47(3), 229–235. 10.1007/PL00001708

[ece370333-bib-0035] Frouz, J. , & Finer, L. (2007). Diurnal and seasonal fluctuations in wood ant (*Formica polyctena*) nest temperature in two geographically distant populations along a south – North gradient. Insectes Sociaux, 54(3), 251–259. 10.1007/s00040-007-0939-4

[ece370333-bib-0036] Frouz, J. , & Jilková, V. (2008). The effect of ants on soil properties and processes (hymenoptera: Formicidae). Myrmecological News, 11(11), 191–199.

[ece370333-bib-0037] Frouz, J. , Jílková, V. , & Sorvari, J. (2016). Contribution of wood ants to nutrient cycling and ecosystem function. In J. A. Stockan & E. J. H. Robinson (Eds.), Wood ant ecology and conservation (pp. 207–220). Cambridge University Press.

[ece370333-bib-0038] Fukasawa, Y. , Osono, T. , & Takeda, H. (2011). Wood decomposing abilities of diverse lignicolous fungi on nondecayed and decayed beech wood. Mycologia, 103(3), 474–482. 10.3852/10-246 21262989

[ece370333-bib-0039] Gherbawy, Y. , Kesselboth, C. , Elhariry, H. , & Hoffmann, K. (2010). Molecular barcoding of microscopic fungi with emphasis on the mucoralean genera *Mucor* and *Rhizopus* . In Y. Gherbawy & K. Voigt (Eds.), Molecular identification of fungi (pp. 213–250). Springer. 10.1007/978-3-642-05042-8_11

[ece370333-bib-0040] Giordano, L. , Gonthier, P. , Varese, G. C. , Miserere, L. , & Nicolotti, G. (2009). Mycobiota inhabiting sapwood of healthy and declining scots pine (*Pinus sylvestris* L.) trees in the Alps. Fungal Diversity, 38(69), e83.

[ece370333-bib-0041] Golubev, V. I. , & Bab'eva, I. P. (1972). *Debaryomyces formicarius* sp. N. And *Debaryomyces cantarellii* associated with the ants of the group *Formica rufa* L. The Journal of General and Applied Microbiology, 18(3), 249–254. 10.2323/jgam.18.249

[ece370333-bib-0042] Gorfer, M. , Mayer, M. , Berger, H. , Rewald, B. , Tallian, C. , Matthews, B. , Sandén, H. , Katzensteiner, K. , & Godbold, D. L. (2021). High fungal diversity but low seasonal dynamics and ectomycorrhizal abundance in a mountain beech Forest. Microbial Ecology, 82(1), 243–256. 10.1007/s00248-021-01736-5 33755773 PMC8282586

[ece370333-bib-0043] Grantina, L. , Bondare, G. , Janberga, A. , Tabors, G. , Kasparinskis, R. , Nikolajeva, V. , & Muiznieks, I. (2012). Monitoring seasonal changes in microbial populations of spruce forest soil of the northern temperate zone. Estonian Journal of Ecology, 61(3), 190. 10.3176/eco.2012.3.03

[ece370333-bib-0044] Gryganskyi, A. P. , Golan, J. , Muszewska, A. , Idnurm, A. , Dolatabadi, S. , Mondo, S. J. , Kutovenko, V. B. , Kutovenko, V. O. , Gajdeczka, M. T. , Anishchenko, I. M. , Pawlowska, J. , Tran, N. V. , Ebersberger, I. , Voigt, K. , Wang, Y. , Chang, Y. , Pawlowska, T. E. , Heitman, J. , Vilgalys, R. , … Spatafora, J. W. (2023). Sequencing the genomes of the first terrestrial fungal lineages: What have we learned? Microorganisms, 11(7), 1830. 10.3390/microorganisms11071830 37513002 PMC10386755

[ece370333-bib-0045] Herrera, H. , Barros‐Parada, W. , & Bergmann, J. (2019). Linoleic acid and stearic acid are biosynthetic precursors of (7 Z, 10 Z)‐7, 10‐hexadecadienal, the major component of the sex pheromone of *Chilecomadia valdiviana* (Lepidoptera: Cossidae). PLoS One, 14(4), e0215769. 10.1371/journal.pone.0215769 31013309 PMC6478319

[ece370333-bib-0046] Hesseltine, C. W. , & Ellis, J. J. (1964). The genus Absidia: Gongronella and cylindrical‐Spored species of Absidia. Mycologia, 56(4), 568–601. 10.1080/00275514.1964.12018145

[ece370333-bib-0047] Hoff, J. A. , Klopfenstein, N. B. , McDonald, G. I. , Tonn, J. R. , Kim, M.‐S. , Zambino, P. J. , Hessburg, P. F. , Rogers, J. D. , Peever, T. L. , & Carris, L. M. (2004). Fungal endophytes in woody roots of Douglas‐fir (*Pseudotsuga menziesii*) and ponderosa pine (*Pinus ponderosa*). Forest Pathology, 34(4), 255–271. 10.1111/j.1439-0329.2004.00367.x

[ece370333-bib-0048] Hoffmann, K. (2010). Identification of the genus Absidia (Mucorales, Zygomycetes): A comprehensive taxonomic revision. In Y. Gherbawy & K. Voigt (Eds.), Molecular identification of fungi (pp. 439–460). Springer Berlin Heidelberg. 10.1007/978-3-642-05042-8_19

[ece370333-bib-0049] Hoffmann, K. , Discher, S. , & Voigt, K. (2007). Revision of the genus Absidia (Mucorales, Zygomycetes) based on physiological, phylogenetic, and morphological characters; thermotolerant Absidia spp. form a coherent group, Mycocladiaceae fam. Nov. Mycological Research, 111(10), 1169–1183. 10.1016/j.mycres.2007.07.002 17997297

[ece370333-bib-0050] Holdenrieder, O. , Baumann, E. , & Schmid‐Haas, P. (1994). Isolation of decay fungi from increment cores: Frustrating experience from Switzerland. *8. International conference on root and butt rots, Uppsala (Sweden), 9–16 Aug 1993*.

[ece370333-bib-0051] Holdenrieder, O. , & Sieber, T. N. (1992). Fungal associations of serially washed healthy non‐mycorrhizal roots of *Picea abies* . Mycological Research, 96(2), 151–156. 10.1016/S0953-7562(09)80932-5

[ece370333-bib-0052] Hölldobler, B. , & Kwapich, C. L. (2022). The guests of ants: How Myrmecophiles interact with their hosts. Harvard University Press.

[ece370333-bib-0053] Hölldobler, B. , & Wilson, E. O. (1990). The ants. Harvard University Press.

[ece370333-bib-0054] Hyde, K. D. , Norphanphoun, C. , Abreu, V. P. , Bazzicalupo, A. , Thilini Chethana, K. W. , Clericuzio, M. , Dayarathne, M. C. , Dissanayake, A. J. , Ekanayaka, A. H. , He, M.‐Q. , Hongsanan, S. , Huang, S.‐K. , Jayasiri, S. C. , Jayawardena, R. S. , Karunarathna, A. , Konta, S. , Kušan, I. , Lee, H. , Li, J. , … Mortimer, P. E. (2017). Fungal diversity notes 603–708: Taxonomic and phylogenetic notes on genera and species. Fungal Diversity, 87(1), 1–235. 10.1007/s13225-017-0391-3

[ece370333-bib-0055] Jílková, V. (2015). Wood ants of genus Formica as important ecosystem engeneers [dissertation thesis]. Institute for Environmental Studies, Charles University.

[ece370333-bib-0056] Jílková, V. , Šebek, O. , & Frouz, J. (2012). Mechanisms of pH change in wood ant (*Formica polyctena*) nests. Pedobiologia, 55(5), 247–251. 10.1016/j.pedobi.2012.04.002

[ece370333-bib-0057] Jouquet, P. , Dauber, J. , Lagerlöf, J. , Lavelle, P. , & Lepage, M. (2006). Soil invertebrates as ecosystem engineers: Intended and accidental effects on soil and feedback loops. Applied Soil Ecology, 32(2), 153–164. 10.1016/j.apsoil.2005.07.004

[ece370333-bib-0058] Jurgensen, M. F. , Finér, L. , Domisch, T. , Kilpeläinen, J. , Punttila, P. , Ohashi, M. , Niemelä, P. , Sundström, L. , Neuvonen, S. , & Risch, A. C. (2008). Organic mound‐building ants: Their impact on soil properties in temperate and boreal forests. Journal of Applied Entomology, 132(4), 266–275. 10.1111/j.1439-0418.2008.01280.x

[ece370333-bib-0059] Käaeik, A. , & Kennebfelt, E. (1957). Investigations on the fungal flora of spruce and pine stumps. Meddelanden Fran Statens Skogsforskningsinstitut, 47(7), 3–88.

[ece370333-bib-0060] Kadochová, Š. (2017). Thermoregulation in ant genus Formica, an individual vs. Colony conflict [Dissertation thesis]. Institute for Environmental Studies, Charles University.

[ece370333-bib-0061] Kernaghan, G. , & Patriquin, G. (2011). Host associations between fungal root endophytes and boreal trees. Microbial Ecology, 62(2), 460–473. 10.1007/s00248-011-9851-6 21475991

[ece370333-bib-0062] Kilpeläinen, J. , Finér, L. , Niemelä, P. , Domisch, T. , Neuvonen, S. , Ohashi, M. , Risch, A. C. , & Sundström, L. (2007). Carbon, nitrogen and phosphorus dynamics of ant mounds (*Formica rufa* group) in managed boreal forests of different successional stages. Applied Soil Ecology, 36(2–3), 156–163. 10.1016/j.apsoil.2007.01.005

[ece370333-bib-0063] Kirk, P. (1999). In R. K. Robinson & C. A. Batt (Eds.), Encyclopedia of food microbiology (pp. 882–887). Academic Press.

[ece370333-bib-0064] Klamer, M. , Lind, A. M. , & Gams, W. (2001). Fungal succession during composting of Miscanthus straw and pig slurry. Acta Horticulturae, 549, 37–46. 10.17660/ActaHortic.2001.549.3

[ece370333-bib-0065] Kovář, P. , Vojtíšek, P. , & Zentsová, I. (2013). Ants as ecosystem engineers in natural restoration of human made habitats. Journal of Landscape Ecology, 6(1), 18–31. 10.2478/v10285-012-0061-9

[ece370333-bib-0066] Kronauer, D. J. , & Pierce, N. E. (2011). Myrmecophiles. Current Biology, 21(6), R208–R209. 21419982 10.1016/j.cub.2011.01.050

[ece370333-bib-0067] Kuhlman, E. G. (1969). Mucorales isolated from pine root bark and wood. Canadian Journal of Botany, 47(11), 1719–1723. 10.1139/b69-249

[ece370333-bib-0068] Kwaśna, H. , Mazur, A. , Łabędzki, A. , Kuźmiński, R. , & Łakomy, P. (2016). Zbiorowiska grzybów w rozkładającym się drewnie dębu i sosny. Leśne Prace Badawcze, 77(3), 261–275. 10.1515/frp-2016-0028

[ece370333-bib-0069] Laakso, J. , & Setälä, H. (1998). Composition and trophic structure of detrital food web in ant Nest mounds of *Formica aquilonia* and in the surrounding Forest soil. Oikos, 81(2), 266–278. 10.2307/3547047

[ece370333-bib-0070] Lindström, S. , Timonen, S. , & Sundström, L. (2021). The bacterial and fungal community composition in time and space in the nest mounds of the ant *Formica exsecta* (hymenoptera: Formicidae). MicrobiologyOpen, 10(4), e1201. 10.1002/mbo3.1201 34459553 PMC8289489

[ece370333-bib-0071] Lindström, S. , Timonen, S. , Sundström, L. , & Johansson, H. (2019). Ants reign over a distinct microbiome in forest soil. Soil Biology and Biochemistry, 139, 107529. 10.1016/j.soilbio.2019.107529

[ece370333-bib-0072] Lindström, S. , Timonen, S. S. , & Sundström, L. (2023). Microbial communities of the ant *Formica exsecta* and its nest material. European Journal of Soil Science, 74(3), e13364. 10.1111/ejss.13364

[ece370333-bib-0073] Linnemann, G. (1941). Die Mucorineen‐Gattung Mortierella Coemans (p. 23). Pflanzenforschung, Heft.

[ece370333-bib-0074] Liu, K. L. , Porras‐Alfaro, A. , Kuske, C. R. , Eichorst, S. A. , & Xie, G. (2012). Accurate, rapid taxonomic classification of fungal large‐subunit rRNA genes. Applied and Environmental Microbiology, 78(5), 1523–1533. 10.1128/AEM.06826-11 22194300 PMC3294464

[ece370333-bib-0075] Lucas, J. M. , Madden, A. A. , Penick, C. A. , Epps, M. J. , Marting, P. R. , Stevens, J. L. , Fergus, D. J. , Dunn, R. R. , & Meineke, E. K. (2019). Azteca ants maintain unique microbiomes across functionally distinct nest chambers. Proceedings of the Royal Society B: Biological Sciences, 286, 20191026. 10.1098/rspb.2019.1026 PMC6710589 31387509

[ece370333-bib-0076] Maavara, V. , Martin, A.‐J. , Oja, A. , & Nuorteva, P. (1994). Sampling of different social categories of red wood ants (Formica s. Str.) for biomonitoring. In B. Markert (Ed.), Environmental sampling for trace analysis (pp. 465–490). Wiley‐Blackwell.

[ece370333-bib-0077] Macias, A. M. , Marek, P. E. , Morrissey, E. M. , Brewer, M. S. , Short, D. P. G. , Stauder, C. M. , Wickert, K. L. , Berger, M. C. , Metheny, A. M. , Stajich, J. E. , Boyce, G. , Rio, R. V. M. , Panaccione, D. G. , Wong, V. , Jones, T. H. , & Kasson, M. T. (2019). Diversity and function of fungi associated with the fungivorous millipede, *Brachycybe lecontii* . Fungal Ecology, 41, 187–197. 10.1016/j.funeco.2019.06.006 31871487 PMC6927558

[ece370333-bib-0078] Mäkipää, R. , Rajala, T. , Schigel, D. , Rinne, K. T. , Pennanen, T. , Abrego, N. , & Ovaskainen, O. (2017). Interactions between soil‐ and dead wood‐inhabiting fungal communities during the decay of Norway spruce logs. The ISME Journal, 11(9), 1964–1974. 10.1038/ismej.2017.57 28430188 PMC5563949

[ece370333-bib-0079] Maksimova, I. A. , Glushakova, A. M. , Kachalkin, A. V. , Chernov, I. Y. , Panteleeva, S. N. , & Reznikova, Z. I. (2016). Yeast communities of *Formica aquilonia* colonies. Microbiology, 85(1), 124–129. 10.1134/S0026261716010045 27301134

[ece370333-bib-0080] Mańka, K. (1964). Próby dalszego udoskonalenia zmodyfikowanej metody Warcupa izolowania grzybów z gleby. Prace Komisji Nauk Rolniczych i Komisji Nauk Leśnych. Poznańskie Towarzystwo Przyjaciół Nauk, 17, 29–45.

[ece370333-bib-0081] Meredith, D. S. (1960). Further observations on fungi inhabiting pine stumps. Annals of Botany, 24(1), 63–78. 10.1093/oxfordjournals.aob.a083689

[ece370333-bib-0082] Meyer, S. T. , Neubauer, M. , Sayer, E. J. , Leal, I. R. , Tabarelli, M. , & Wirth, R. (2013). Leaf‐cutting ants as ecosystem engineers: Topsoil and litter perturbations around Atta cephalotes nests reduce nutrient availability. Ecological Entomology, 38(5), 497–504. 10.1111/een.12043

[ece370333-bib-0083] Millar, C. S. (2012). Decomposition of coniferous leaf litter. Biology of Plant Litter Decomposition, 1, 105–128.

[ece370333-bib-0084] Mohamed, H. , El‐Shanawany, R. , Shah, A. M. , Nazir, Y. , Naz, T. , Ullah, S. , Mustafa, K. , & Song, Y. (2019). Comparative analysis of different isolated oleaginous Mucoromycota fungi for their γ‐Linolenic acid and carotenoid production. BioMed Research International, 2020(1), 3621543. 10.1155/2020/3621543 PMC7665918 33204691

[ece370333-bib-0085] Mueller, U. G. , Rehner, S. A. , & Schultz, T. R. (1998). The evolution of agriculture in ants. Science, 281(5385), 2034–2038. 10.1126/science.281.5385.2034 9748164

[ece370333-bib-0086] Muszewska, A. , Okrasińska, A. , Steczkiewicz, K. , Drgas, O. , Orłowska, M. , Perlińska‐Lenart, U. , Aleksandrzak‐Piekarczyk, T. , Szatraj, K. , Zielenkiewicz, U. , Piłsyk, S. , Malc, E. , Mieczkowski, P. , Kruszewska, J. S. , Bernat, P. , & Pawłowska, J. (2021). Metabolic potential, ecology and presence of associated bacteria is reflected in genomic diversity of Mucoromycotina. Frontiers in Microbiology, 12, 636986. 10.3389/fmicb.2021.636986 33679672 PMC7928374

[ece370333-bib-0087] Naranjo‐Ortiz, M. A. , & Gabaldón, T. (2019). Fungal evolution: Diversity, taxonomy and phylogeny of the fungi. Biological Reviews, 94(6), 2101–2137. 10.1111/brv.12550 31659870 PMC6899921

[ece370333-bib-0088] Nel, W. J. , De Beer, Z. W. , Wingfield, M. J. , Poulsen, M. , Aanen, D. K. , Wingfield, B. D. , & Duong, T. A. (2021). Phylogenetic and phylogenomic analyses reveal two new genera and three new species of ophiostomatalean fungi from termite fungus combs. Mycologia, 113(6), 1199–1217. 10.1080/00275514.2021.1950455 34477494

[ece370333-bib-0089] Nepel, M. , Voglmayr, H. , Blatrix, R. , Longino, J. T. , Fiedler, K. , Schönenberger, J. , & Mayer, V. E. (2016). Ant‐cultivated Chaetothyriales in hollow stems of myrmecophytic Cecropia sp. trees–diversity and patterns. Fungal Ecology, 23, 131–140. 10.1016/j.funeco.2016.07.007

[ece370333-bib-0090] Nepel, M. , Voglmayr, H. , Schönenberger, J. , & Mayer, V. E. (2014). High diversity and low specificity of chaetothyrialean fungi in carton galleries in a neotropical ant–plant association. PLoS One, 9(11), e112756. 10.1371/journal.pone.0112756 25398091 PMC4232418

[ece370333-bib-0091] Nguyen, T. T. , Santiago, A. L. C. M. D. A. , Kirk, P. M. , & Lee, H. B. (2023). Discovery of a new Lichtheimia (Lichtheimiaceae, Mucorales) from invertebrate niche and its phylogenetic status and physiological characteristics. Journal of Fungi, 9(3), 317. 10.3390/jof9030317 36983485 PMC10056009

[ece370333-bib-0092] O'Donnell, K. (1993). Fusarium and its near relatives. In The fungal holomorph: Mitotic, meiotic and pleomorphic speciation in fungal systematics (pp. 225–233). CAB International.

[ece370333-bib-0093] Oh, S.‐Y. , Park, M. S. , & Lim, Y. W. (2019). The influence of microfungi on the mycelial growth of ectomycorrhizal fungus *Tricholoma matsutake* . Microorganisms, 7(6), 169. 10.3390/microorganisms7060169 31181710 PMC6617177

[ece370333-bib-0094] Okrasińska, A. , Bokus, A. , Duk, K. , Gęsiorska, A. , Sokołowska, B. , Miłobędzka, A. , Wrzosek, M. , & Pawłowska, J. (2021). New endohyphal relationships between mucoromycota and burkholderiaceae representatives. Applied and Environmental Microbiology, 87(7), e02707‐20. 10.1128/AEM.02707-20 33483310 PMC8091615

[ece370333-bib-0095] Oksanen, J. F. , Blanchet, G. , Friendly, M. , KIndt, R. , Legendre, P. , McGlinn, D. , Minchin, P. R. , O'Hara, R. B. , Simpson, G. L. , Solymos, P. , Stevens, M. H. H. , Szoecs, E. , & Wagner, H. (2019). *Vegan: Community ecology package*. (R package version 2.5‐6.) [computer software]. https://CRAN.R‐project.org/package=vegan

[ece370333-bib-0096] Osono, T. , Hirose, D. , & Fujimaki, R. (2006). Fungal colonization as affected by litter depth and decomposition stage of needle litter. Soil Biology and Biochemistry, 38(9), 2743–2752. 10.1016/j.soilbio.2006.04.028

[ece370333-bib-0097] Osono, T. , Ono, Y. , & Takeda, H. (2003). Fungal ingrowth on forest floor and decomposing needle litter of *Chamaecyparis obtusa* in relation to resource availability and moisture condition. Soil Biology and Biochemistry, 35(11), 1423–1431. 10.1016/S0038-0717(03)00236-0

[ece370333-bib-0098] Parker, J. , & Grimaldi, D. A. (2014). Specialized Myrmecophily at the ecological dawn of modern ants. Current Biology, 24(20), 2428–2434. 10.1016/j.cub.2014.08.068 25283779

[ece370333-bib-0099] Parmentier, T. , Dekoninck, W. , & Wenseleers, T. (2014). A highly diverse microcosm in a hostile world: A review on the associates of red wood ants (*Formica rufa* group). Insectes Sociaux, 61(3), 229–237. 10.1007/s00040-014-0357-3

[ece370333-bib-0100] Pawłowska, J. , Okrasińska, A. , Kisło, K. , Aleksandrzak‐Piekarczyk, T. , Szatraj, K. , Dolatabadi, S. , & Muszewska, A. (2019). Carbon assimilation profiles of mucoralean fungi show their metabolic versatility. Scientific Reports, 9(1), 11864. 10.1038/s41598-019-48296-w 31413281 PMC6694110

[ece370333-bib-0101] Qu, Z.‐L. , Santalahti, M. , Köster, K. , Berninger, F. , Pumpanen, J. , Heinonsalo, J. , & Sun, H. (2021). Soil fungal community structure in boreal pine forests: From southern to subarctic areas of Finland. Frontiers in Microbiology, 12, 653896. 10.3389/fmicb.2021.653896 34122368 PMC8188478

[ece370333-bib-0102] R Core Team . (2020). R: A language and environment for statistical computing [computer software]. R Foundation for Statistical Computing. https://www.R‐project.org/

[ece370333-bib-0103] Rehner, S. A. , & Samuels, G. J. (1994). Taxonomy and phylogeny of Gliocladium analysed from nuclear large subunit ribosomal DNA sequences. Mycological Research, 98(6), 625–634. 10.1016/S0953-7562(09)80409-7

[ece370333-bib-0104] Rim, S. O. , Roy, M. , Jeon, J. , Montecillo, J. A. V. , Park, S.‐C. , & Bae, H. (2021). Diversity and communities of fungal endophytes from four Pinus species in Korea. Forests, 12(3), 302. 10.3390/f12030302

[ece370333-bib-0105] Rodrigues, R. C. , & Fernandez‐Lafuente, R. (2010). Lipase from *Rhizomucor miehei* as a biocatalyst in fats and oils modification. Journal of Molecular Catalysis B: Enzymatic, 66(1–2), 15–32. 10.1016/j.molcatb.2010.03.008

[ece370333-bib-0106] RStudio Team . (2020). RStudio: Integrated development for R. [Computer software]. RStudio. PBC. http://www.rstudio.com/

[ece370333-bib-0107] Ruiz‐González, M. X. , Malé, P.‐J. G. , Leroy, C. , Dejean, A. , Gryta, H. , Jargeat, P. , Quilichini, A. , & Orivel, J. (2010). Specific, non‐nutritional association between an ascomycete fungus and Allomerus plant‐ants. Biology Letters, 7(3), 475–479. 10.1098/rsbl.2010.0920 21084334 PMC3097849

[ece370333-bib-0108] Santalahti, M. , Sun, H. , Jumpponen, A. , Pennanen, T. , & Heinonsalo, J. (2016). Vertical and seasonal dynamics of fungal communities in boreal scots pine forest soil. FEMS Microbiology Ecology, 92(11), fiw170. 10.1093/femsec/fiw170 27515733

[ece370333-bib-0109] Scherba, G. (1958). Reproduction, nest orientation and population structure of an aggregation of mound nests of *Formica ulkei* Emery («Formicidae»). Insectes Sociaux, 5(2), 201–213. 10.1007/BF02224070

[ece370333-bib-0110] Schoch, C. L. , Seifert, K. A. , Huhndorf, S. , Robert, V. , Spouge, J. L. , Levesque, C. A. , Chen, W. , Fungal Barcoding Consortium , Fungal Barcoding Consortium Author List , Bolchacova, E. , Voigt, K. , Crous, P. W. , Miller, A. N. , Wingfield, M. J. , Aime, M. C. , An, K.‐D. , Bai, F.‐Y. , Barreto, R. W. , … White, M. M. (2012). Nuclear ribosomal internal transcribed spacer (ITS) region as a universal DNA barcode marker for fungi. Proceedings of the National Academy of Sciences, 109(16), 6241–6246. 10.1073/pnas.1117018109 PMC3341068 22454494

[ece370333-bib-0111] Sewell, G. W. F. (1959). Studies of fungi in a Calluna‐heathland soil: I. Vertical distribution in soll and on root surfaces. Transactions of the British Mycological Society, 42(3), 343–353. 10.1016/S0007-1536(56)80043-0

[ece370333-bib-0112] Siedlecki, I. , Gorczak, M. , Okrasińska, A. , & Wrzosek, M. (2021). Chance or necessity—The fungi Co‐occurring with *Formica polyctena* ants. Insects, 12(3), 204. 10.3390/insects12030204 33670956 PMC7997191

[ece370333-bib-0113] Siedlecki, I. , Kochanowski, M. , Majchrowska, M. , Błocka, Z. , & Wrzosek, M. (2022). Mycobiota of Formica polyctena ants' infrabuccal pellets—Hidden diversity. IUSSI 2002, International Union for the Study of social insects.

[ece370333-bib-0114] Siedlecki, I. , Piątek, M. , Majchrowska, M. , Okrasińska, A. , Owczarek‐Kościelniak, M. , & Pawłowska, J. (2023). Discovery of formicomyces microglobosus gen. Et sp. Nov. strengthens the hypothesis of independent evolution of ant‐associated fungi in Trichomeriaceae. Fungal Biology, 127(12), 1466–1474. 10.1016/j.funbio.2023.10.005 38097320

[ece370333-bib-0115] Skelton, J. , Jusino, M. A. , Li, Y. , Bateman, C. , Thai, P. H. , Wu, C. , Lindner, D. L. , & Hulcr, J. (2018). Detecting symbioses in complex communities: The fungal symbionts of bark and ambrosia beetles within Asian pines. Microbial Ecology, 76, 839–850. 10.1007/s00248-018-1154-8 29476344

[ece370333-bib-0116] Skirgiełło, A. , Zadara, M. , & Ławrynowicz, M. (1979). Grzyby (Mycota), Tom X, Glonowce (Phycomycetes), Pleśniakowe (Mucorales), Kłębiankowe (Endogonales) (p. 321). PWN.

[ece370333-bib-0117] Söderström, B. E. (1975). Vertical distribution of microfungi in a spruce forest soil in the south of Sweden. Transactions of the British Mycological Society, 65(3), 419–425. 10.1016/S0007-1536(75)80039-8

[ece370333-bib-0118] Sokołowska, B. , Orłowska, M. , Okrasińska, A. , Piłsyk, S. , Pawłowska, J. , & Muszewska, A. (2023). What can be lost? Genomic perspective on the lipid metabolism of Mucoromycota. IMA Fungus, 14(1), 22. 10.1186/s43008-023-00127-4 37932857 PMC10629195

[ece370333-bib-0119] Song, J. , Tang, Z. , Zhao, X. , Yin, Y. , Li, X. , Chen, F. , Chen, A. , & Liu, Y. (2023). Red imported fire ant nesting affects the structure of soil microbial community. Frontiers in Cellular and Infection Microbiology, 13, 1221996. 10.3389/fcimb.2023.1221996 37483389 PMC10358852

[ece370333-bib-0120] Sorvari, J. , & Hartikainen, S. (2021). Terpenes and fungal biomass in the nest mounds of Formica aquilonia wood ants. European Journal of Soil Biology, 105, 103336. 10.1016/j.ejsobi.2021.103336

[ece370333-bib-0121] Stockan, J. A. , & Robinson, E. J. (2016). Wood ant ecology and conservation. Cambridge University Press.

[ece370333-bib-0122] Summerbell, R. C. (2005). Root endophyte and mycorrhizosphere fungi of black spruce, *Picea mariana*, in a boreal forest habitat: Influence of site factors on fungal distributions. Studies in Mycology, 53(1), 121–145. 10.3114/sim.53.1.121

[ece370333-bib-0123] Tedersoo, L. , Bahram, M. , Põlme, S. , Kõljalg, U. , Yorou, N. S. , Wijesundera, R. , Ruiz, L. V. , Vasco‐Palacios, A. M. , Thu, P. Q. , Suija, A. , Smith, M. E. , Sharp, C. , Saluveer, E. , Saitta, A. , Rosas, M. , Riit, T. , Ratkowsky, D. , Pritsch, K. , Põldmaa, K. , … Abarenkov, K. (2014). Global diversity and geography of soil fungi. Science, 346(6213), 1256688. 10.1126/science.1256688 25430773

[ece370333-bib-0124] Telagathoti, A. , Probst, M. , & Peintner, U. (2021). Habitat, snow‐cover and soil pH, affect the distribution and diversity of mortierellaceae species and their associations to bacteria. Frontiers in Microbiology, 12, 669784. 10.3389/fmicb.2021.669784 34276602 PMC8283828

[ece370333-bib-0125] Terhonen, E. , Keriö, S. , Sun, H. , & Asiegbu, F. O. (2014). Endophytic fungi of Norway spruce roots in boreal pristine mire, drained peatland and mineral soil and their inhibitory effect on *Heterobasidion parviporum* in vitro. Fungal Ecology, 9, 17–26. 10.1016/j.funeco.2014.01.003

[ece370333-bib-0126] Toju, H. , & Sato, H. (2018). Root‐associated fungi shared between arbuscular mycorrhizal and ectomycorrhizal conifers in a temperate forest. Frontiers in Microbiology, 9, 342426. 10.3389/fmicb.2018.00433 PMC5858530 29593682

[ece370333-bib-0127] Travanty, N. V. , Vargo, E. L. , Apperson, C. S. , & Ponnusamy, L. (2022). Colonization by the red imported fire ant, *Solenopsis invicta* modifies soil bacterial communities. Microbial Ecology, 84(1), 240–256. 10.1007/s00248-021-01826-4 34370055

[ece370333-bib-0128] Turner, M. , & Pugh, G. J. F. (1961). Species of Mortierella from a salt marsh. Transactions of the British Mycological Society, 44(2), 243‐IN9.

[ece370333-bib-0129] Vandepol, N. , Liber, J. , Desirò, A. , Na, H. , Kennedy, M. , Barry, K. , Grigoriev, I. V. , Miller, A. N. , O'Donnell, K. , & Stajich, J. E. (2020). Resolving the Mortierellaceae phylogeny through synthesis of multi‐gene phylogenetics and phylogenomics. Fungal Diversity, 104, 1–23. 10.1007/s13225-020-00455-5 PMC7751987 33364917

[ece370333-bib-0130] Vilgalys, R. , & Hester, M. (1990). Rapid genetic identification and mapping of enzymatically amplified ribosomal DNA from several Cryptococcus species. Journal of Bacteriology, 172(8), 4238–4246. 10.1128/jb.172.8.4238-4246.1990 2376561 PMC213247

[ece370333-bib-0131] Villanueva, R. A. M. , & Chen, Z. J. (2019). ggplot2: Elegant graphics for data analysis (2nd ed.). Measurement: Interdisciplinary Research and Perspectives, 17(3), 160–167. 10.1080/15366367.2019.1565254

[ece370333-bib-0132] Voglmayr, H. , Mayer, V. , Maschwitz, U. , Moog, J. , Djieto‐Lordon, C. , & Blatrix, R. (2011). The diversity of ant‐associated black yeasts: Insights into a newly discovered world of symbiotic interactions. Fungal Biology, 115(10), 1077–1091. 10.1016/j.funbio.2010.11.006 21944219

[ece370333-bib-0133] Voigt, K. , James, T. Y. , Kirk, P. M. , Santiago, A. L. C. M. D. A. , Waldman, B. , Griffith, G. W. , Fu, M. , Radek, R. , Strassert, J. F. H. , Wurzbacher, C. , Jerônimo, G. H. , Simmons, D. R. , Seto, K. , Gentekaki, E. , Hurdeal, V. G. , Hyde, K. D. , Nguyen, T. T. T. , & Lee, H. B. (2021). Early‐diverging fungal phyla: Taxonomy, species concept, ecology, distribution, anthropogenic impact, and novel phylogenetic proposals. Fungal Diversity, 109, 59–98. 10.1007/s13225-021-00480-y 34608378 PMC8480134

[ece370333-bib-0134] Vu, D. , Groenewald, M. , De Vries, M. , Gehrmann, T. , Stielow, B. , Eberhardt, U. , Al‐Hatmi, A. , Groenewald, J. Z. , Cardinali, G. , Houbraken, J. , Boekhout, T. , Crous, P. W. , & Verkley, G. J. M. (2019). Large‐scale generation and analysis of filamentous fungal DNA barcodes boosts coverage for kingdom fungi and reveals thresholds for fungal species and higher taxon delimitation. Studies in Mycology, 92(1), 135–154. 10.1016/j.simyco.2018.05.001 29955203 PMC6020082

[ece370333-bib-0135] Wagner, L. , Stielow, B. , Hoffmann, K. , Petkovits, T. , Papp, T. , Vágvölgyi, C. , De Hoog, G. , Verkley, G. , & Voigt, K. (2013). A comprehensive molecular phylogeny of the Mortierellales (Mortierellomycotina) based on nuclear ribosomal DNA. Persoonia: Molecular Phylogeny and Evolution of Fungi, 30(17), 77–93. 10.3767/003158513X666268 PMC3734968 24027348

[ece370333-bib-0136] Walther, G. , Pawłowska, J. , Alastruey‐Izquierdo, A. , Wrzosek, M. , Rodriguez‐Tudela, J. L. , Dolatabadi, S. , Chakrabarti, A. , & de Hoog, G. S. (2013). DNA barcoding in Mucorales: An inventory of biodiversity. Persoonia: Molecular Phylogeny and Evolution of Fungi, 30(37), 11–47. 10.3767/003158513X665070 PMC3734965 24027345

[ece370333-bib-0137] Wang, Y.‐N. , Liu, X.‐Y. , & Zheng, R.‐Y. (2022). The *Umbelopsis ramanniana* Sensu Lato consists of five cryptic species. Journal of Fungi, 8(9), 895. 10.3390/jof8090895 36135620 PMC9506118

[ece370333-bib-0138] Watanabe, T. (2002). Pictorial atlas of soil and seed fungi: Morphologies of cultured fungi and key to species. CRC Press.

[ece370333-bib-0139] Watanabe, T. , Watanabe, Y. , Fukatsu, T. , & Kurane, R. (1998). *Mortierella isabellina* and *M. lignicola* from decayed wood with termite nests in Yakushima, Japan. Mycoscience, 39(4), 475–476. 10.1007/BF02460909

[ece370333-bib-0140] Wells, R. L. , Murphy, S. K. , & Moran, M. D. (2017). Habitat modification by the leaf‐cutter ant, Atta cephalotes, and patterns of leaf‐litter arthropod communities. Environmental Entomology, 46(6), 1264–1274. 10.1093/ee/nvx162 29126135

[ece370333-bib-0141] White, T. J. , Bruns, T. , Lee, S. , & Taylor, J. (1990). Amplification and direct sequencing of fungal ribosomal RNA genes for phylogenetics. PCR Protocols: A Guide to Methods and Applications, 18(1), 315–322.

[ece370333-bib-0142] Williams, J. , McKay, S. , Khalfan, M. , Hilgert, U. , Lauter, S. , & Jeong, E. S. (2014). DNA Subway–an educational bioinformatics platform for gene and genome analysis: DNA barcoding, and RNA‐Seq. *Proceedings of the 10th world congress on genetics applied to livestock production, Vancouver, BC, Canada*, 17–22.

[ece370333-bib-0143] Wills, B. D. , & Landis, D. A. (2018). The role of ants in north temperate grasslands: A review. Oecologia, 186(2), 323–338. 10.1007/s00442-017-4007-0 29147779 PMC5799350

[ece370333-bib-0144] Yegin, S. , Fernandez‐Lahore, M. , Jose Gama Salgado, A. , Guvenc, U. , Goksungur, Y. , & Tari, C. (2011). Aspartic proteinases from Mucor spp. in cheese manufacturing. Applied Microbiology and Biotechnology, 89, 949–960. 10.1007/s00253-010-3020-6 21127856

[ece370333-bib-0145] Zettler, J. A. , Mcinnis, T. M., Jr. , Allen, C. R. , & Spira, T. P. (2002). Biodiversity of fungi in red imported fire ant (hymenoptera: Formicidae) mounds. Annals of the Entomological Society of America, 95(4), 487–491. 10.1603/0013-8746(2002)095[0487:BOFIRI]2.0.CO;2

[ece370333-bib-0146] Zhu, G. , Ding, W. , Xue, M. , Zhao, Y. , Li, M. , & Li, Z. (2022). Identification and pathogenicity of a new entomopathogenic fungus, *Mucor hiemalis* (Mucorales: Mucorales), on the root maggot, *Bradysia odoriphaga* (Diptera: Sciaridae). Journal of Insect Science, 22(2), 2. 10.1093/jisesa/ieac010 PMC8932411 35303105

[ece370333-bib-0147] Zong, T.‐K. , Zhao, H. , Liu, X.‐L. , Ren, L.‐Y. , Zhao, C.‐L. , & Liu, X.‐Y. (2021). Taxonomy and phylogeny of four new species in Absidia (Cunninghamellaceae, Mucorales) from China. Frontiers in Microbiology, 12, 677836. 10.3389/fmicb.2021.677836 34421840 PMC8371387

